# Multi-view attention-based deep learning for benign–malignant classification of pulmonary nodules

**DOI:** 10.1371/journal.pone.0357963

**Published:** 2026-09-15

**Authors:** Lijun Zhang, Debing Zhuo, Xiaolu Wu, Yuchou He, Guodong Kang

**Affiliations:** 1 School of Computer Science and Engineering, Jishou University, Zhangjiajie, Hunan, China; 2 College of Intelligent Construction, Jishou University, Zhangjiajie, Hunan, China; University of Electronic Science and Technology of China, CHINA

## Abstract

Accurate classification of pulmonary nodules is essential for early lung cancer diagnosis, yet the limited spatial information provided by a single computed tomography slice may hinder the characterization of complex nodule morphology. Although multi-view strategies can capture complementary anatomical information, conventional fusion methods often fail to account for lesion-specific features and differences in the contribution of individual views. To address these limitations, we propose a tri-view pulmonary nodule classification framework that combines Lesion-Guided Attention (LGA) with View-Weighted Fusion (VWF). The framework extracts complementary representations from axial, coronal, and sagittal slices. LGA introduces lesion masks as weak spatial priors to refine lesion-related feature responses while preserving contextual information, whereas VWF learns sample-dependent weights to adaptively aggregate the three view representations. The framework was evaluated on 869 nodules (448 benign and 421 malignant) derived from the public Lung Image Database Consortium and Image Database Resource Initiative (LIDC-IDRI) dataset. It achieved an accuracy of 93.08%, an AUC of 97.06%, and an F1-score of 92.68%, representing the highest values for these three metrics among the models included in the baseline comparison. Ablation analysis further showed that the combined use of LGA and VWF provided the best overall balance among the evaluated configurations. In the displayed test cases, Gradient-weighted Class Activation Mapping visualizations showed that the learned responses were concentrated mainly within or adjacent to lesion-relevant regions. These findings support the potential value of combining lesion-aware feature refinement with adaptive multi-view fusion for pulmonary nodule classification within the evaluated LIDC-IDRI cohort.

## Introduction

### Background and motivation

Lung cancer remains one of the leading causes of cancer-related mortality worldwide. According to the 2020 global cancer estimates, approximately 2.2 million new cases and 1.8 million deaths occur annually [1]. Prognosis is strongly associated with the stage at diagnosis, and early detection can substantially improve patient survival [2]. Pulmonary nodules are common early radiological manifestations of lung cancer; therefore, accurate benign–malignant classification is important for screening, treatment planning, and follow-up management.

Computed tomography (CT), particularly low-dose CT (LDCT), has been widely used for lung cancer screening and has demonstrated benefits in early-stage detection and mortality reduction [3,4]. However, the interpretation of pulmonary nodules still depends heavily on radiologists’ experience and is affected by subjectivity, limited reproducibility, and increasing workload. Computer-aided diagnosis methods may assist radiologists by providing consistent assessments and visual evidence for pulmonary nodule evaluation [5].

Deep learning has achieved substantial progress in pulmonary nodule analysis. Convolutional neural networks (CNNs) can automatically learn texture, shape, and boundary features from CT images [5,6], while Transformer-based and CNN–Transformer hybrid architectures provide broader contextual modeling through self-attention [7,8]. Nevertheless, some existing methods rely primarily on two-dimensional slices, which may not adequately represent morphological variations across different spatial directions [6].

Multi-view strategies provide a practical means of incorporating complementary spatial information without the computational burden of full three-dimensional (3D) modeling [9,10]. However, simple concatenation or fixed-weight fusion cannot adapt to differences in the diagnostic information provided by individual views. In addition, attention mechanisms are often learned implicitly and may respond to irrelevant background regions rather than lesion-related features [11]. Although three-dimensional models can capture volumetric information, they generally require greater computational resources and larger training datasets [5,12]. Therefore, effectively combining lesion-oriented feature refinement with adaptive multi-view fusion remains an important problem in pulmonary nodule classification.

To address these limitations, we propose a tri-view pulmonary nodule classification framework based on axial, coronal, and sagittal CT slices. The use of tri-planar input alone is not considered the methodological novelty of this study because similar representations have been investigated previously [13]. Instead, the principal contribution lies in the coordinated integration of Lesion-Guided Attention (LGA) and View-Weighted Fusion (VWF). LGA uses lesion masks as weak spatial priors to refine lesion-related features while preserving contextual information, whereas VWF learns sample-dependent, Softmax-normalized weights to adaptively aggregate the three view representations. The framework is evaluated through comparisons with commonly used single-view backbones, module ablation experiments, and qualitative Grad-CAM visualization.

### Related work and challenges

#### Convolutional neural network-based pulmonary nodule benign–malignant classification.

CNNs have been widely applied to pulmonary nodule classification because of their ability to learn local texture, shape, and edge features directly from CT images [5,6]. Representative studies, including the NoduleX framework, demonstrated that deep CNN features can provide effective malignancy prediction on the LIDC-IDRI dataset [14].

To incorporate spatial information beyond a single slice, several studies have introduced three-dimensional CNNs. Dou et al. developed multi-level contextual 3D CNNs for pulmonary nodule analysis [15], while Hussein et al. employed a 3D CNN-based multi-task framework for nodule risk stratification [16]. Multi-scale three-dimensional networks have also been investigated to improve nodule representation [17]. Although these methods capture volumetric characteristics, their computational and data requirements may limit their use in relatively small medical imaging datasets.

#### Multi-view and multi-slice modeling approaches.

Multi-view and multi-slice methods have been developed to represent the three-dimensional characteristics of pulmonary nodules using complementary two-dimensional images [9,10]. These methods extract information from multiple slices or anatomical directions and generally provide richer spatial representations than single-view approaches.

Chang et al. proposed a multiview residual selective kernel network using axial, coronal, and sagittal CT views together with handcrafted texture features [13]. This work represents a closely related tri-planar classification strategy. Unlike that approach, the present framework uses lesion masks as weak spatial priors for soft residual feature refinement and learns sample-dependent view contributions through Softmax-normalized weighting. Other collaborative or multi-task approaches have also demonstrated the value of combining complementary nodule representations [16,18]. However, adaptive integration of lesion-refined features across views remains insufficiently investigated.

#### Transformer-based approaches for medical image analysis.

Vision Transformers have been introduced into medical image analysis because self-attention can model long-range dependencies and global semantic relationships [19]. Vision Transformer (ViT) and Swin Transformer provide representative self-attention-based architectures for image recognition [20,21], and Transformer-based variants have increasingly been investigated in medical imaging applications [8,19]. However, pure Transformer models generally require substantial training data, and their performance may be less stable when labeled medical datasets are limited [19].

#### CNN–transformer hybrid architectures and attention mechanisms.

CNN–Transformer hybrid architectures combine the local feature extraction capability of CNNs with the contextual modeling capability of Transformers. Medical Vision Transformer (MedViT) integrates efficient convolutional modules and Transformer blocks and has demonstrated strong performance across several medical image classification tasks [22], providing the backbone architecture used in this study.

Attention mechanisms can further enhance responses to informative regions, while Gradient-weighted Class Activation Mapping (Grad-CAM) provides qualitative visualization of class-related activations [11,23]. Nevertheless, implicitly learned attention does not necessarily correspond to lesion regions [24]. This limitation motivates the introduction of explicit lesion guidance together with adaptive multi-view fusion in the proposed framework.

## Materials and methods

### Methodology

#### Overall model architecture.

**Fig 1:**
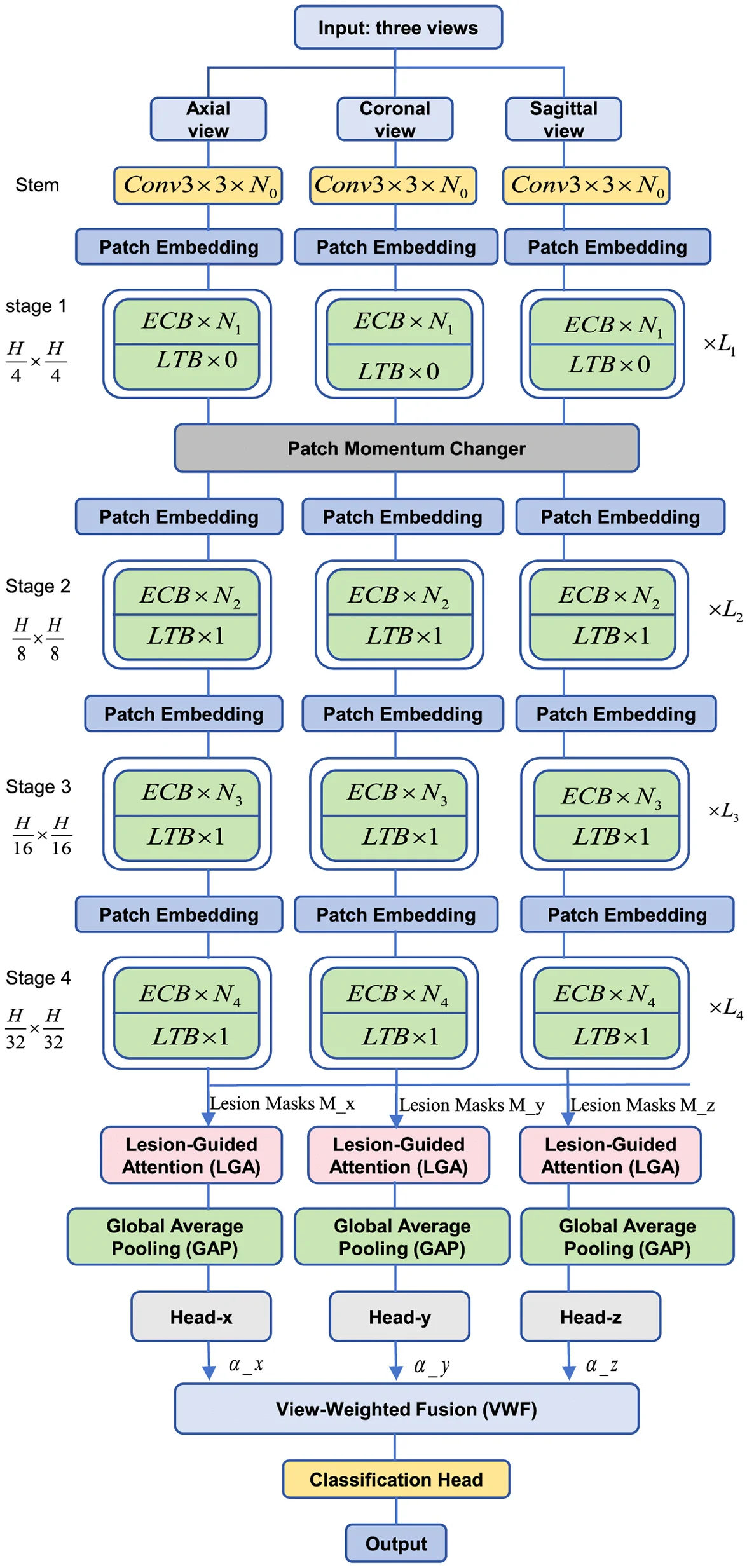
Overall architecture of the proposed tri-view pulmonary nodule classification model. Representative axial, coronal, and sagittal slices are processed by independent MedViT branches. High-level features are refined by the Lesion-Guided Attention (LGA) module using lesion masks as weak spatial priors and then aggregated by the View-Weighted Fusion (VWF) module using sample-dependent weights. The fused representation is passed to the classifier for benign–malignant prediction.

This study develops a tri-view model for pulmonary nodule benign–malignant classification using MedViT as the encoder backbone, as illustrated in Fig 1. Representative axial, coronal, and sagittal slices are processed by independent encoding branches, refined using LGA, and adaptively aggregated using VWF before classification.

#### Tri-view input encoding module.

To construct the tri-view input, a consensus mask is first generated from the LIDC-IDRI radiologist annotations using a 50% agreement criterion. A 50 × 50 × 50-voxel cube centered at the centroid of this consensus mask is then cropped. This size includes the nodule and limited perinodular context while restricting irrelevant background and computational cost. The slice with the largest lesion-mask area is selected from each axial, coronal, and sagittal plane and resized to 224 × 224; HU-based intensity normalization is described in the Dataset and preprocessing section. Let v∈{x,y,z} denote the axial, coronal, and sagittal views, respectively.

Each slice is processed by a MedViT branch with an identical architecture and output dimension but non-shared trainable parameters. In MedViT, Efficient Convolution Blocks (ECBs) primarily model local features, Local Transformer Blocks (LTBs) provide contextual feature modeling, and the Patch Momentum Changer (PMC) serves as a feature-regularization module [22]. Specifically, the Patch Embedding parameters, the convolutional and normalization parameters in the ECBs, and the attention, projection, and normalization parameters in the LTBs are maintained separately for the three branches. Let θx, θy, and θz denote the corresponding parameter sets. These branches are jointly optimized under the same classification objective, whereas the Patch Momentum Changer (PMC) is shared across views. At the l-th stage, the feature map of view v is expressed as:


$$\begin{array}{c} F_{v}^{\left(l\right)}\in R^{C_{l}\times H_{l}\times W_{l}} \end{array}$$

where C_l_ denotes the channel dimension, whereas H_l_ and W_l_ denote the spatial dimensions of the feature map at stage l, respectively.

In the multi-view parallel encoding setting, feature distributions may vary across different views, which can negatively affect subsequent cross-view fusion. To mitigate inter-view distribution discrepancies, the Patch Momentum Changer (PMC) module from the original MedViT architecture is retained and applied after the first encoding stage [22]. The PMC module performs feature regularization by perturbing and reorganizing the statistical properties of mini-batch features, which was intended to smooth feature distributions before subsequent cross-view fusion [22]. In the implementation, PMC was applied only during training, with an activation probability of 0.5 and a mixing strength of 0.5. The same batch permutation and mixing coefficient were applied to the three view branches, whereas PMC was disabled during validation and testing. The transformation can be formulated as:


$$\begin{array}{c} {\overset{\sim }{F}}_{v}^{\left(\text{1}\right)}=PMC\left(F_{v}^{\left(\text{1}\right)}\right) \end{array}$$

where ${\overset{\sim }{F}}_{v}^{\left(\text{1}\right)}$ denotes the feature representation after PMC processing.

Table 1 summarizes the input size, encoder configuration, parameter-sharing scheme, and output dimension of each view branch.

**Table 1:** Tri-view input configuration and feature dimensions of the proposed framework.

View	Input Size	Encoder	Shared Modules	Output Dimension
**Axial (x-view)**	224 × 224	MedViT Encoder	PMC	256
**Coronal (y-view)**	224 × 224	MedViT Encoder	PMC	256
**Sagittal (z-view)**	224 × 224	MedViT Encoder	PMC	256

^a^Each view uses an identical MedViT encoder; “PMC” denotes the shared module across views.

^b^“Output dimension” indicates the feature channel dimension of the encoder output.

^c^The three MedViT encoder branches have identical architectures but separate trainable parameter sets ($\theta_{x}$, $\theta_{y}$, and $\theta_{z}$), including the Patch Embedding, ECB, and LTB parameters; only the PMC module is shared across views.

**Fig 2:**
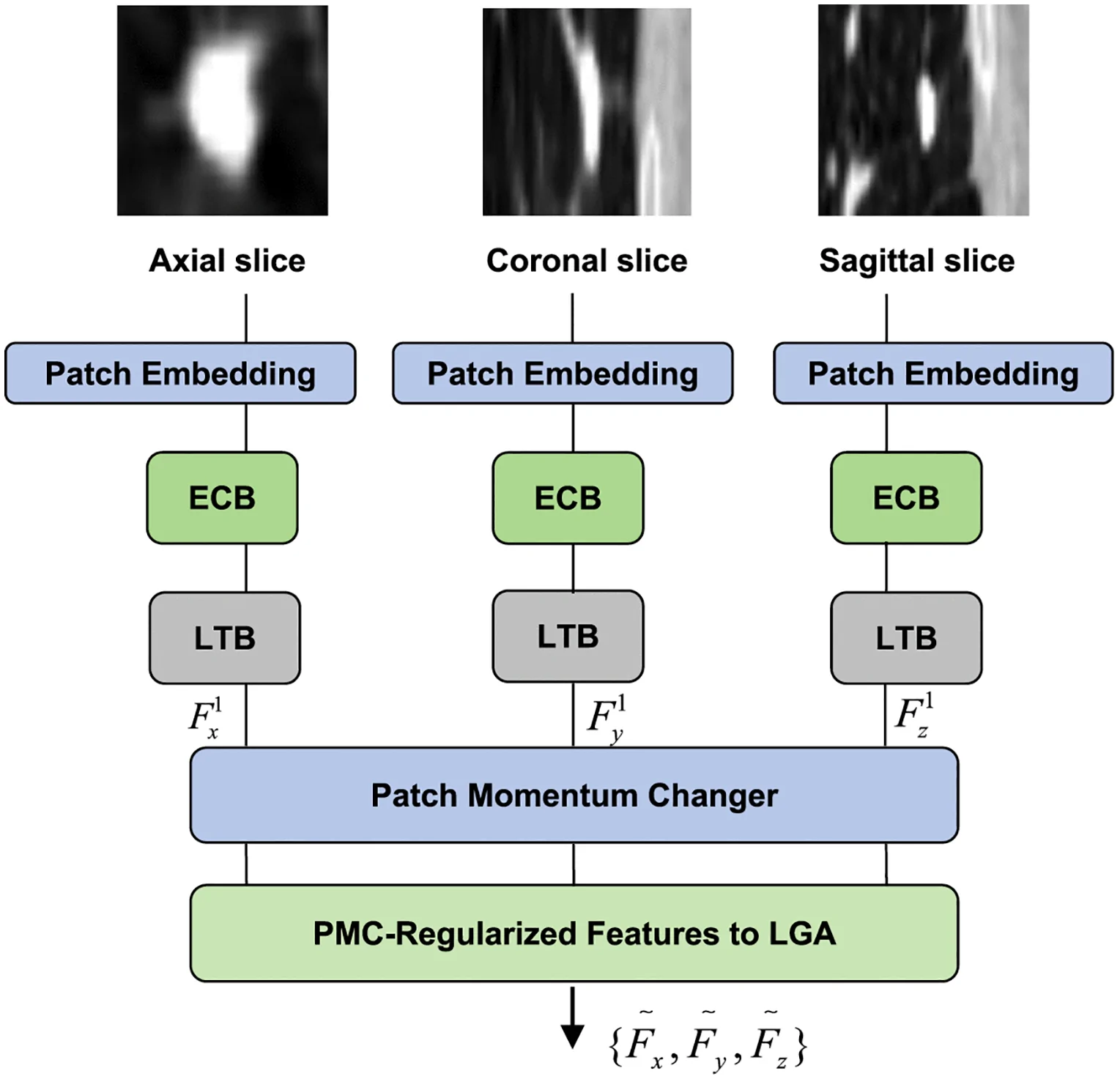
Tri-view parallel encoding strategy. Representative axial, coronal, and sagittal slices are processed by three encoder branches with identical architectures and non-shared trainable parameters. Each branch adopts MedViT as the encoder, whereas the Patch Momentum Changer (PMC) is shared across the branches after the first encoding stage. The resulting view-specific features are subsequently passed to the LGA module.

The tri-view encoding process is illustrated in Fig 2. The resulting view-specific features are subsequently refined by LGA before adaptive fusion.

#### Lesion-Guided Attention module.

In pulmonary nodule benign–malignant classification, the nodule region typically occupies only a small portion of the CT image and is characterized by complex density variations and blurred boundaries. As a result, conventional convolutional or Transformer-based feature extraction processes may allocate excessive attention to irrelevant background regions, which can adversely affect classification performance and model interpretability [5]. To address this issue, a Lesion-Guided Attention (LGA) module is introduced to explicitly guide the spatial attention distribution by integrating lesion location priors with feature responses [24].

The LGA module takes as input the feature maps $F_{v}$ from the tri-view encoding module, where v∈{x,y,z} denotes the axial, coronal, and sagittal views, respectively, along with the corresponding binary lesion masks Mv. The lesion masks used in all experiments were generated during preprocessing from the LIDC-IDRI radiologist annotations using a 50% consensus criterion and served as weak spatial priors for attention modulation [24,25]. No additional segmentation network was trained as part of the present classification pipeline.

The lesion masks are not used for hard cropping or hard masking. Each branch receives the same nodule-centered patch, and the aligned mask is introduced only after high-level feature extraction to generate attention weights. The residual pathway preserves the original representation while selectively enhancing lesion-related responses, thereby retaining limited perinodular context that may also be informative for classification [26]. This design is intended to reduce dependence on a single exact lesion boundary when radiologist delineations differ or nodule margins are indistinct [25].

Specifically, each binary lesion mask is replicated along the channel dimension and spatially aligned to the spatial resolution of the corresponding feature map before feature modulation. This operation yields an aligned guidance mask that is compatible with the feature map for element-wise multiplication. For notational simplicity, the mask symbol used in the following equations refers to this aligned guidance mask rather than to the original binary mask. The lesion-guided attention weight map is then computed as:


$$\begin{array}{c} A_{\nu }=\sigma \left(W_{\text{2}}*ReLU\left(W_{\text{1}}*\left(F_{\nu }⊙M_{\nu }\right)\right)\right), \end{array}$$

where $W_{\text{1}}$ and $W_{\text{2}}$ denote learnable 1 × 1 convolution kernels, * represents convolution, ⊙ denotes element-wise multiplication, σ(·) is the Sigmoid function, and ReLU denotes the rectified linear unit activation. This operation generates a soft lesion-guided attention map from the masked feature responses. In the implementation, W_1_ projected the 256-channel input feature map to 64 hidden channels, whereas W_2_ projected the resulting representation to a single-channel attention map.

The guided feature enhancement is then performed as


$$\begin{array}{c} F_{v}^{\prime }=F_{v}⊙\left(\text{1}+\lambda A_{v}\right), \end{array}$$

where λ is a fixed scaling factor that controls the strength of lesion-guided spatial modulation and was set to 0.5 in all experiments. The identity term prevents complete suppression of the baseline feature response.

To control the influence of strong prior guidance and support stable feature integration, a residual enhancement pathway is incorporated into the LGA module before output fusion:


$$\begin{array}{c} F_{v}^{out}=F_{v}+\alpha \cdot F_{v}^{\prime }, \end{array}$$

where α is a learnable residual scaling parameter initialized to 0.1 and jointly optimized with the remaining network parameters. Thus, λ controls the strength of spatial modulation, whereas α controls the contribution of the enhanced residual branch and is intended to limit excessive reliance on the lesion prior.

LGA adds mask resizing and spatial alignment, element-wise operations, and two 1 × 1 convolutions after backbone feature extraction. A dedicated runtime or floating-point-operation comparison with region-of-interest cropping was not performed; therefore, no computational superiority is claimed. Application to data without pre-existing lesion masks would require an upstream segmentation source.

**Fig 3:**
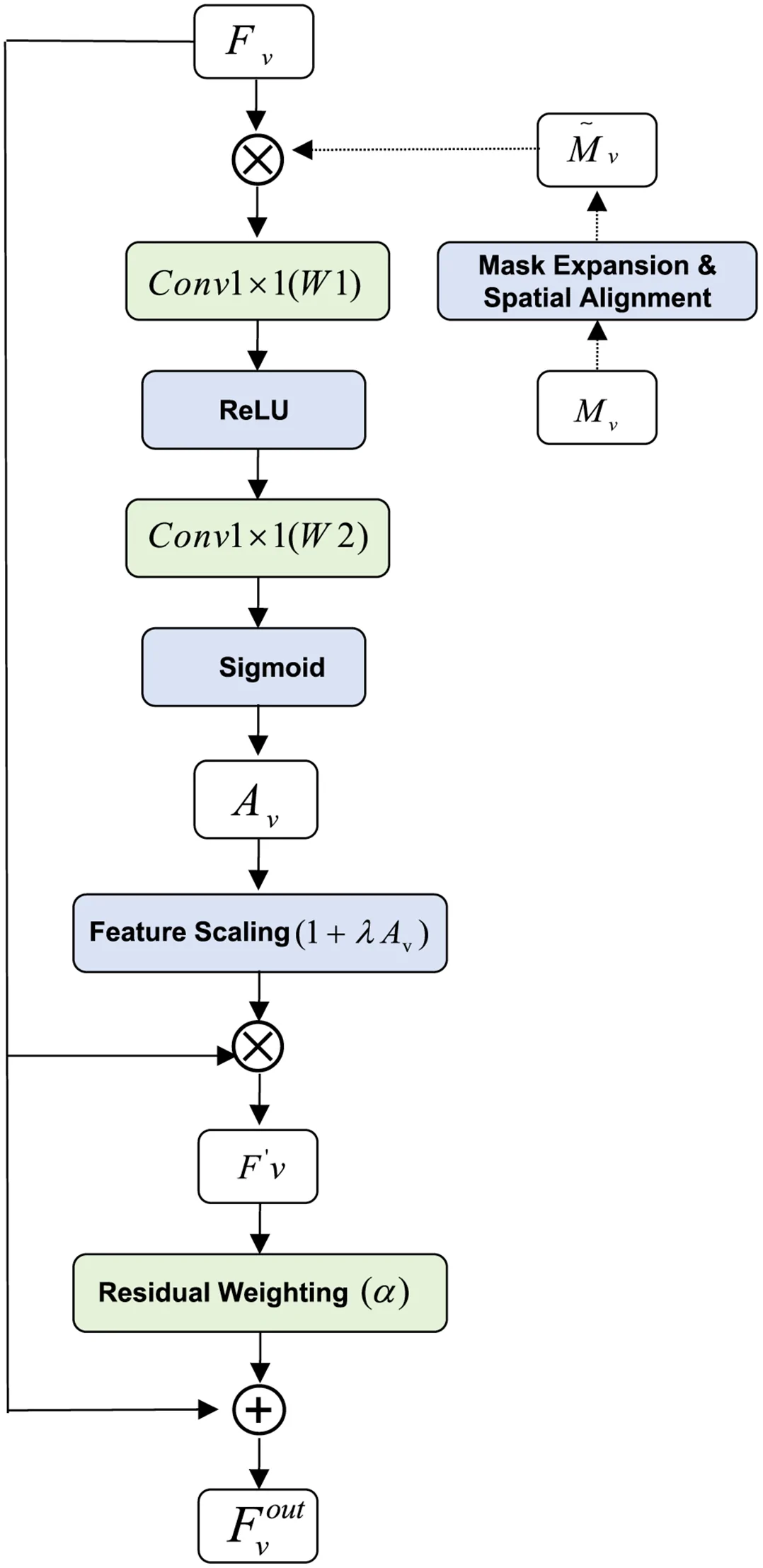
Architecture of the Lesion-Guided Attention (LGA) module. The lesion mask is expanded and spatially aligned with the corresponding feature map. Mask-guided responses are processed by two 1 × 1 convolutions with rectified linear unit activation and a Sigmoid function to generate the attention map. The enhanced features are combined with the original representation through a learnable residual scaling coefficient and subsequently passed to VWF.

The complete LGA processing flow is illustrated in Fig 3. Its output features are subsequently supplied to VWF for cross-view aggregation.

#### Learnable View-Weighted Fusion module.

**Fig 4:**
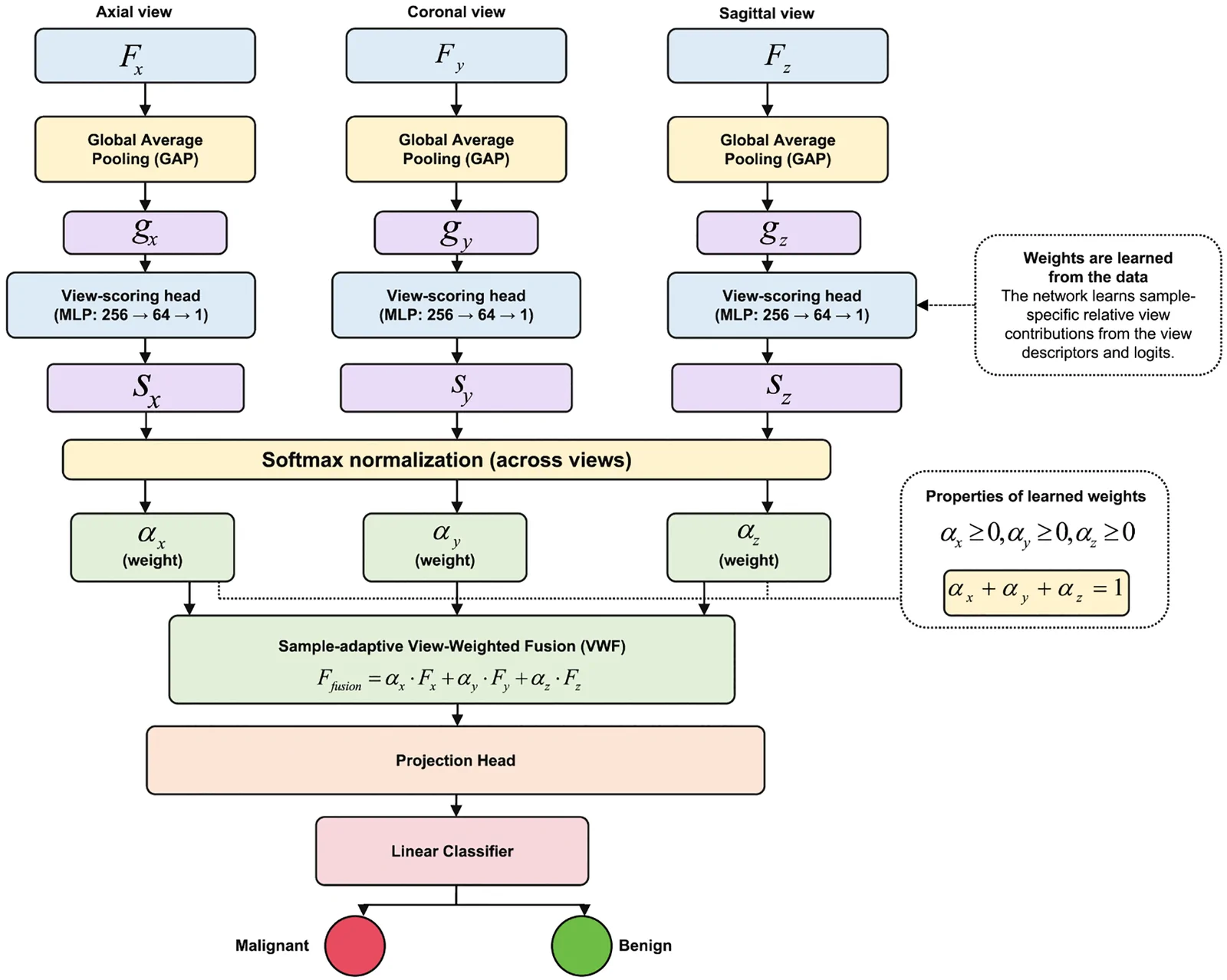
Sample-adaptive View-Weighted Fusion (VWF) module. The LGA-enhanced feature maps from the axial, coronal, and sagittal views are globally average-pooled to obtain view-specific descriptors. A learnable view-scoring head produces three unnormalized logits, which are jointly normalized using Softmax to generate sample-dependent weights $\alpha_{x}$, $\alpha_{y}$, and $\alpha_{z}$. The weights are non-negative and satisfy $\alpha_{x}+\alpha_{y}+\alpha_{z}=\text{1}$. The fused representation is obtained through weighted aggregation of the three view features and is subsequently forwarded to the projection head and classifier for benign–malignant prediction.

After LGA, the axial, coronal, and sagittal features contain complementary information but may contribute unequally to individual nodules. The VWF module therefore generates one score from each view descriptor and jointly normalizes the three scores using Softmax. The resulting weights are sample-dependent, non-negative, and sum to one, enabling end-to-end weighted aggregation without explicit cross-view attention interactions. The VWF processing flow is illustrated in Fig 4.

Let v∈{x,y,z} denote the view index corresponding to the axial, coronal, and sagittal views. For notational simplicity, $F_{v}$ in this section denotes the LGA output feature map $F_{v}^{out}\in {\mathbb{R}}^{C\times H\times W}$ defined above. VWF first applies global average pooling (GAP) to obtain a compact view descriptor:


$$\begin{array}{c} g_{v}=GAP\left(F_{v}\right)\in R^{C} \end{array}$$

A lightweight learnable view-scoring head is then used to produce an unnormalized view logit $s_{v}$ for each view:


$$\begin{array}{c} s_{v}=\varphi (g_{v}),v\in \{x,y,z\} \end{array}$$

where φ denotes a two-layer multilayer perceptron with dimensions 256 → 64 → 1 and a ReLU activation between the two linear layers. Separate scoring heads with the same architecture were used for the axial, coronal, and sagittal views. The three logits are jointly normalized by Softmax for each nodule:


$$\begin{array}{c} \alpha_{v}=\frac{\text{exp}(s_{v})}{\sum_{u\in \{x,y,z\}}\text{exp}(s_{u})},v\in \{x,y,z\} \end{array}$$

The three coefficients are recalculated for each nodule and are jointly optimized with the encoders, LGA, and classifier. They represent model-internal relative view contributions rather than manually assigned clinical importance scores, and the mechanism is conceptually related to adaptive expert gating [27].

Finally, the fused feature representation $F_{fusion}$ is computed by weighted aggregation:


$$\begin{array}{c} F_{fusion}=\sum_{v\in \{x,y,z\}}\alpha_{v}F_{v}. \end{array}$$

The resulting fused representation was subsequently passed through a 256-dimensional projection head, followed by a ReLU activation and a dropout layer with a rate of 0.3. A single-unit linear classifier then produced the malignancy logit.

#### Backbone building blocks (ECB and LTB).

To balance representational capacity and computational efficiency in medical imaging settings, we adopt the Efficient Convolution Block (ECB) and the Local Transformer Block (LTB) from the original MedViT framework as the fundamental feature extraction units [22]. It should be noted that we do not modify the internal architectures or computational procedures of ECB and LTB; detailed designs and implementation specifics can be found in the original MedViT paper [22]. This section provides further details on these building blocks to facilitate understanding of the overall model architecture.

The ECB combines depthwise separable convolutions with Multi-Head Convolutional Attention, enabling efficient modeling of local spatial patterns with a relatively low computational budget [22]. By leveraging multi-scale convolutional operations, ECB captures local contextual cues in nodule regions, while this attention mechanism models responses across channels and spatial locations, further enhancing local feature representations in an efficient manner [22].

The LTB introduces an Efficient Self-Attention mechanism to model long-range dependencies with controlled complexity [22]. Specifically, this mechanism projects the input feature map into Query, Key, and Value embeddings and applies multi-head self-attention to capture global contextual information. The resulting features are then refined through pointwise convolution and linear mappings to produce the output representation. In MedViT, the LTB complements the ECB by providing global context support for higher-level semantic representations [22].

In the proposed framework, ECB and LTB form the four-stage MedViT backbone, while LGA and VWF operate on the resulting high-level view features.

#### Loss function.

To optimize the proposed model for pulmonary nodule benign–malignant classification, we used the standard binary cross-entropy (BCE) objective. Let z_i_ ∈ R denote the output logit for the i-th sample, p_i_ = σ(z_i_) the corresponding predicted probability of malignancy, and y_i_∈{0,1} the ground-truth label, where 1 denotes malignant and 0 denotes benign. The BCE loss is expressed as:


$$\begin{array}{c} L_{BCE}=-\frac{\text{1}}{N}\sum_{i=\text{1}}^{N}\left[y_{i}log(p_{i})+(\text{1}-y_{i})log(\text{1}-p_{i})\right] \end{array}$$

where N denotes the number of samples in a mini-batch. In implementation, the raw logits were directly passed to PyTorch’s BCEWithLogitsLoss, which combines the Sigmoid operation and BCE calculation in a numerically stable form. No class weights were applied. Regularization was introduced through the decoupled weight decay of the AdamW optimizer rather than through an explicit penalty term added to the loss [28]. The weight decay coefficient was set to 0.05.

### Experimental setup

#### Dataset and preprocessing.

Experiments were conducted on the publicly available Lung Image Database Consortium and Image Database Resource Initiative (LIDC-IDRI) dataset, which contains 1,018 thoracic CT scans [25]. The dataset was obtained from The Cancer Imaging Archive (TCIA) (https://doi.org/10.7937/K9/TCIA.2015.LO9QL9SX).

Nodules identified by at least three radiologists were retained. The malignancy-labeling rule described by Chang et al. [13] was adopted. Each available radiologist assigned a malignancy score ranging from 1 to 5, and the median of the available ratings was used as the final score for each nodule. Nodules with a median score below 3 were labeled as benign, those with a median score above 3 were labeled as malignant, and those with a median score equal to 3 were regarded as indeterminate and excluded from the binary classification task. After excluding nodules for which valid consensus masks or complete tri-view inputs could not be generated, the final cohort comprised 869 nodules, including 448 benign and 421 malignant nodules.

These labels were derived from radiologist-assigned malignancy ratings rather than pathological confirmation and were therefore treated as operational labels for the present study. Excluding nodules with a median score of 3 reduced label ambiguity but restricted the analysis to a relatively less ambiguous subset of LIDC-IDRI.

**Fig 5:**
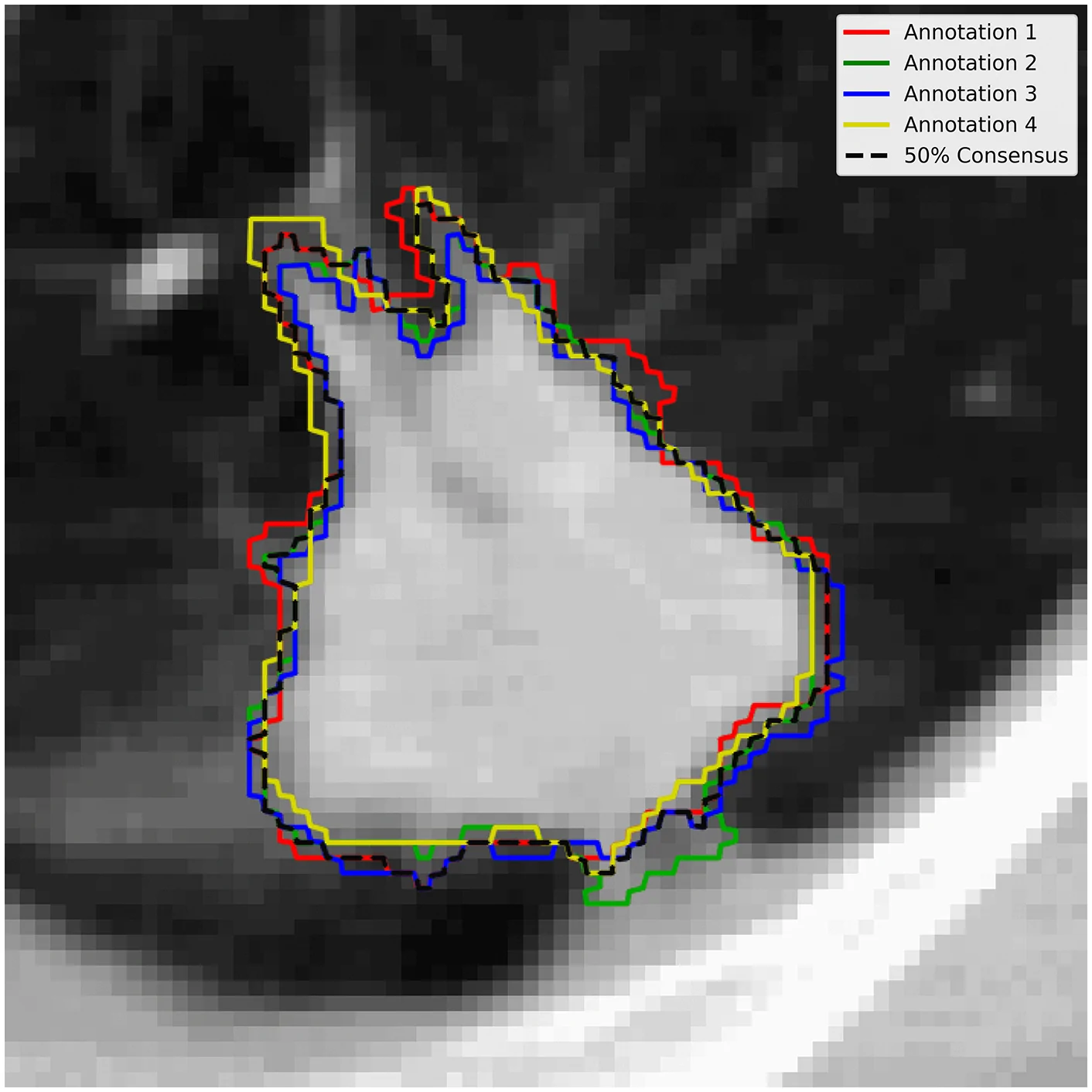
Visualization of radiologist annotations and consensus-derived segmentation for a representative pulmonary nodule using LIDC-IDRI data. Colored solid contours indicate the nodule boundaries independently delineated by four radiologists (red: Annotation 1; green: Annotation 2; blue: Annotation 3; yellow: Annotation 4). The black dashed contour represents the boundary of the 50%-agreement consensus mask. The consensus region captures the overlapping portion of the radiologist annotations and was used to construct the lesion mask for subsequent processing.

Fig 5 provides a representative visualization of the individual radiologist annotations and the corresponding consensus mask.

The 869 nodules were divided at the nodule level into training, validation, and test sets using stratified random sampling at an approximate ratio of 70:15:15, yielding 609 training nodules, 130 validation nodules, and 130 test nodules. The corresponding benign/malignant distributions were 314/295, 67/63, and 67/63, respectively. All axial, coronal, and sagittal images and lesion masks derived from the same nodule were assigned to the same subset. Because partitioning was performed at the nodule level rather than the patient level, distinct nodules from the same patient could be assigned to different subsets. Therefore, the protocol prevented nodule-level overlap but did not enforce complete patient-level independence. Unless otherwise specified, all performance metrics were calculated and reported at the nodule level.

**Fig 6:**
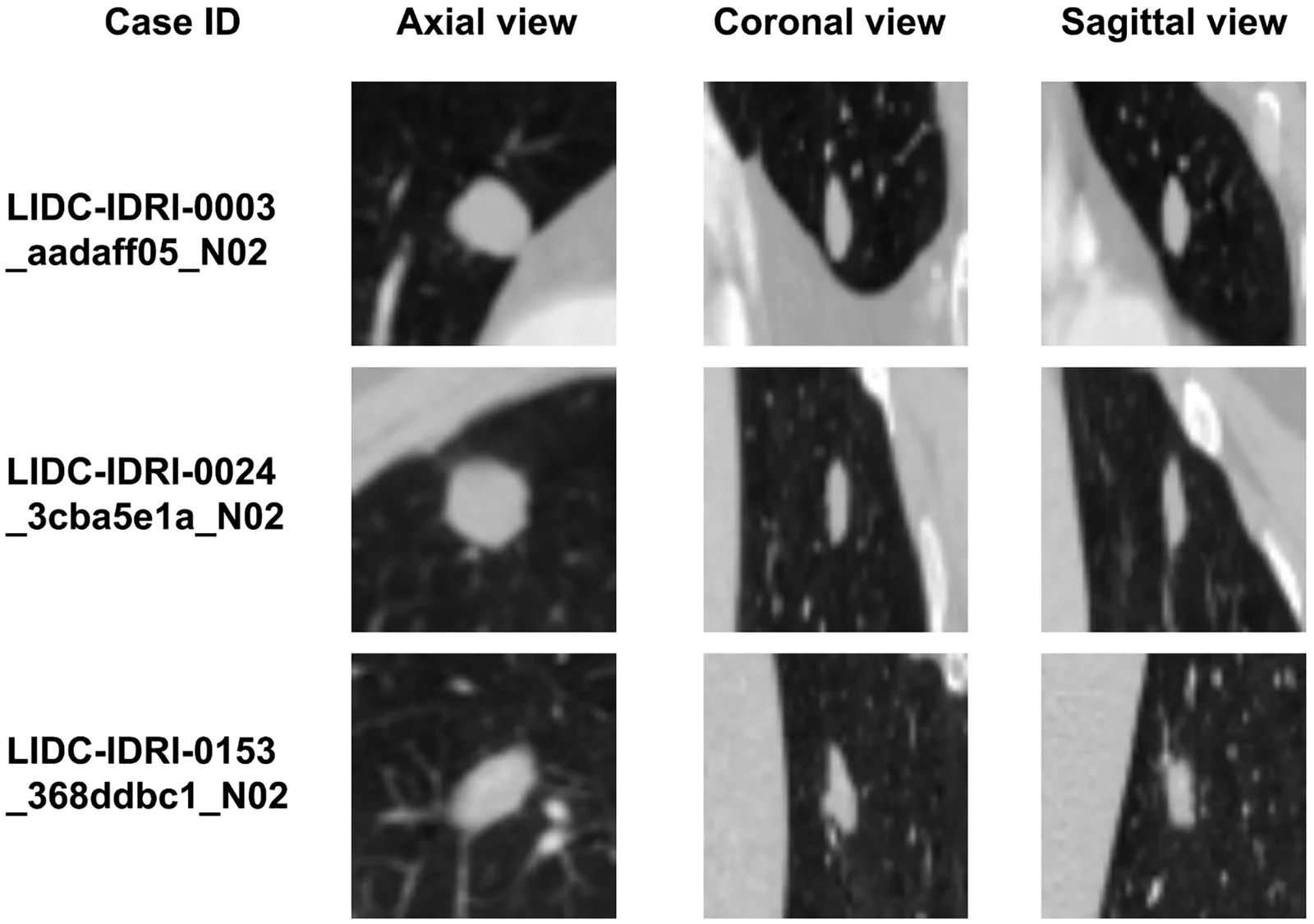
Representative tri-view pulmonary nodule examples from the LIDC-IDRI dataset.

The CT volumes were processed on their native voxel grids without isotropic spatial resampling. For each eligible nodule, a fixed-size 50 × 50 × 50-voxel patch was cropped around the centroid of the consensus mask. From each patch, the slice with the largest lesion-mask area was selected in each of the axial, coronal, and sagittal directions rather than the geometrical middle slice. The CT slices were resized to 224 × 224 using bilinear interpolation, whereas the corresponding binary masks were resized using nearest-neighbor interpolation. Fig 6 provides representative examples of tri-view nodule images, highlighting the complementary anatomical information captured by different planes.

To reduce intensity variability caused by different scanners and reconstruction settings, all images are normalized based on Hounsfield Units (HU). Specifically, intensity values are clipped to the range [−1000, 400] HU and then linearly rescaled to the range [0, 1] to provide a stable input distribution for feature learning. Each normalized grayscale slice was then replicated across three channels to provide an RGB-compatible input for the encoder networks. To enhance generalization, we applied on-the-fly (online) data augmentation during training. Random transformations were generated when each training sample was loaded rather than being pre-generated and stored as additional image files. Therefore, the same original nodule could undergo different transformations across training epochs. For each nodule, synchronized two-dimensional geometric transformations were applied to the axial, coronal, and sagittal images and their corresponding masks within each training iteration. These transformations included small rotations (±7°), translations (≤5%), scaling (0.95–1.05×), random horizontal flipping (probability 0.5), and random cropping retaining 90–100% of the image area. Mild photometric perturbations, including gamma correction (0.9–1.1) and contrast adjustment (0.9–1.1), were applied only to the CT images. During validation and testing, no random augmentation was applied; only deterministic preprocessing was performed.

Training samples were randomly shuffled at each epoch using the standard data-loading procedure. No class-balanced sampler, class reweighting, or class-weighted loss was applied because the training subset was approximately balanced.

#### Experimental settings.

Data preprocessing was performed on a local workstation running Windows 10 using Python version 3.9.8. The LIDC-IDRI data were accessed and organized using pylidc version 0.2.3 and pydicom version 2.4.4. Core numerical and image-processing operations were implemented using NumPy version 2.0.2, pandas version 2.3.3, OpenCV (opencv-python) version 5.0.0.93, and Pillow version 11.3.0. Model development, training, and inference were conducted on an AutoDL workstation equipped with a single NVIDIA graphics processing unit with 32 GB of memory. The model environment used Python version 3.10.8, PyTorch version 2.1.2, torchvision version 0.16.2, and einops version 0.8.2, with CUDA 12.1 support. To support reproducibility, the random seed for model training and data loading was fixed to 42. Data loading used eight worker processes with pinned memory enabled.

During inference, the three preprocessed views of each nodule were jointly processed as a single sample to generate one benign–malignant probability for nodule-level evaluation.

All models were trained from scratch without ImageNet or other pretrained weights. Model parameters were initialized using the default initialization routines of the corresponding PyTorch modules, and no additional global initialization procedure was applied. AdamW was used with an initial learning rate of 5 × 10−5 and a weight decay of 0.05. The learning rate was linearly increased from 1 × 10 − 6–5 × 10 − 5 during the first five epochs and was subsequently reduced to a minimum of 1 × 10 − 6 using cosine decay. Training was performed for up to 30 epochs with a batch size of 16, and the gradient norm was clipped at 1.0. Early stopping was triggered if the validation AUC did not improve for 10 consecutive epochs, and the checkpoint with the highest validation AUC was retained for test-set evaluation.

For baseline comparison, ResNet50 [29], DenseNet121 [30], EfficientNet-B3 [31], ViT[20], Swin Transformer [21], and MedViT[22] were trained and evaluated using the same nodule-level data partition, preprocessing pipeline, augmentation settings, loss function, model-selection criterion, and test-set evaluation procedure as the proposed model. Each baseline model used the representative axial slice as the single-view input and was trained without pretrained weights.

After the checkpoint with the highest validation AUC was selected, the classification threshold was determined on the validation set by maximizing the Youden index and was then applied unchanged to the test set. AUC was calculated from the continuous prediction probabilities.

### Ablation study design

To assess the contribution of each component, five configurations were evaluated: (1) Baseline, a single-view MedViT model; (2) MV, a tri-view model without LGA or VWF; (3) MV + VWF; (4) MV + LGA; and (5) Full, which incorporated MV, LGA, and VWF. In the MV and MV + LGA configurations, the three view features were combined by direct concatenation, whereas VWF was used only in configurations explicitly marked with VWF. All configurations used the same nodule-level data partition, preprocessing pipeline, loss function, model-selection criterion, and evaluation protocol. Accuracy (ACC), AUC, and F1-score were used as the primary metrics for the ablation analysis.

### Visualization and interpretability analysis

To qualitatively examine the spatial evidence associated with model predictions, Grad-CAM was applied to the single-view MedViT baseline and the full model [23]. Heatmaps were generated for the predicted class using a predefined high-level feature layer and resized to the corresponding input-image resolution. Selected nodules from the held-out test set were used to compare the activation patterns of the two models. The same preprocessing and inference procedures used for test-set evaluation were applied. The heatmaps were used only for qualitative inspection of lesion-relevant activation patterns and were not interpreted as quantitative localization metrics or evidence of clinical validity.

### Evaluation metrics

Classification performance was evaluated using accuracy (ACC), area under the receiver operating characteristic curve (AUC), sensitivity (recall), specificity, and F1-score.

Accuracy (ACC) measures the proportion of correctly classified samples among all samples and is defined as:


$$\begin{array}{c} ACC=\frac{TP+TN}{TP+TN+FP+FN} \end{array}$$

where TP, TN, FP, and FN denote true positives, true negatives, false positives, and false negatives, respectively, with malignant nodules treated as the positive class. Although the class proportions were closely matched across the training, validation, and test sets through stratified sampling, ACC and F1-score were interpreted together with sensitivity, specificity, and AUC because threshold-dependent metrics can be influenced by class prevalence.

Sensitivity (also referred to as Recall) evaluates the detection capability for malignant nodules, i.e., the proportion of true malignant cases correctly identified as malignant:


$$\begin{array}{c} Sensitivity=\frac{TP}{TP+FN} \end{array}$$

Specificity measures the ability to correctly reject benign nodules, i.e., the proportion of true benign cases correctly classified as benign:


$$\begin{array}{c} Specificity=\frac{TN}{TN+FP} \end{array}$$

Sensitivity and specificity were interpreted jointly because they reflect complementary errors involving false negatives and false positives.

To balance Precision and Recall, we further report the F1-score, defined as the harmonic mean of Precision and Recall:


$$\begin{array}{c} F\text{1}=\frac{\text{2}TP}{\text{2}TP+FP+FN} \end{array}$$

where Precision is the proportion of predicted malignant nodules that are truly malignant:


$$\begin{array}{c} Precision=\frac{TP}{TP+FP} \end{array}$$

Finally, we use AUC to quantify the overall discriminative ability of the model across different decision thresholds. Unlike threshold-dependent metrics, AUC provides a threshold-free assessment and is widely adopted for binary classification in medical imaging studies [5].

Because malignant nodules were treated as the positive class, sensitivity was interpreted together with ACC, specificity, F1-score, and AUC.

### Statistical analysis

Statistical analyses were performed in Python version 3.10.8 using scikit-learn version 1.7.2 [32]. AUC was calculated from the continuous malignancy probabilities. For threshold-dependent metrics, the decision threshold was selected on the validation set by maximizing the Youden index, defined as sensitivity plus specificity minus one, and was subsequently applied unchanged to the held-out test set. ACC, sensitivity, specificity, and F1-score were calculated using this fixed threshold. To quantify uncertainty in the test-set performance estimates, 95% confidence intervals (CIs) were calculated using 5,000 stratified bootstrap resamples while preserving the benign and malignant class counts. The validation-derived decision threshold was kept fixed during bootstrap resampling, and the 2.5th and 97.5th percentiles were used as the CI bounds. No hypothesis testing or multiple-comparison procedures were performed.

### Ethics statement

This study performed a secondary analysis of the publicly available, de-identified LIDC-IDRI dataset obtained from The Cancer Imaging Archive (TCIA). No human participants were recruited, and no identifiable private information was accessed; therefore, institutional review board approval and informed consent were not required.

## Results

### Comparison with baseline models

The test-set performance of the proposed model and baseline models is summarized in Table 2.

**Table 2:** Test-set performance comparison of the proposed multi-view model and baseline models on the LIDC-IDRI dataset.

Model	View	ACC(%)	AUC(%)	F1(%)	Sensitivity (%)	Specificity (%)
**ResNet50**	Single	90.00	96.02	89.26	85.71	94.03
**DenseNet121**	Single	90.77	96.52	90.16	87.30	94.03
**EfficientNet-B3**	Single	86.15	95.38	87.14	**96.83**	76.12
**ViT**	Single	77.69	82.92	78.20	82.54	73.13
**Swin Transformer**	Single	77.69	85.79	75.63	71.43	83.58
**MedViT**	Single	92.31	94.65	91.80	88.89	**95.52**
**Ours**	Multi-view	**93.08**	**97.06**	**92.68**	90.48	**95.52**

^a^“Single-view” denotes models trained with a single 2D slice (axial view); “Multi-view” denotes the proposed tri-view setting (axial/coronal/sagittal).

^b^Area under the receiver operating characteristic curve (AUC) is threshold-independent.

^c^Best point estimates are highlighted in bold.

^d^Bootstrap-derived 95% confidence intervals for all evaluated models are provided in S2 Dataset.

**Fig 7:**
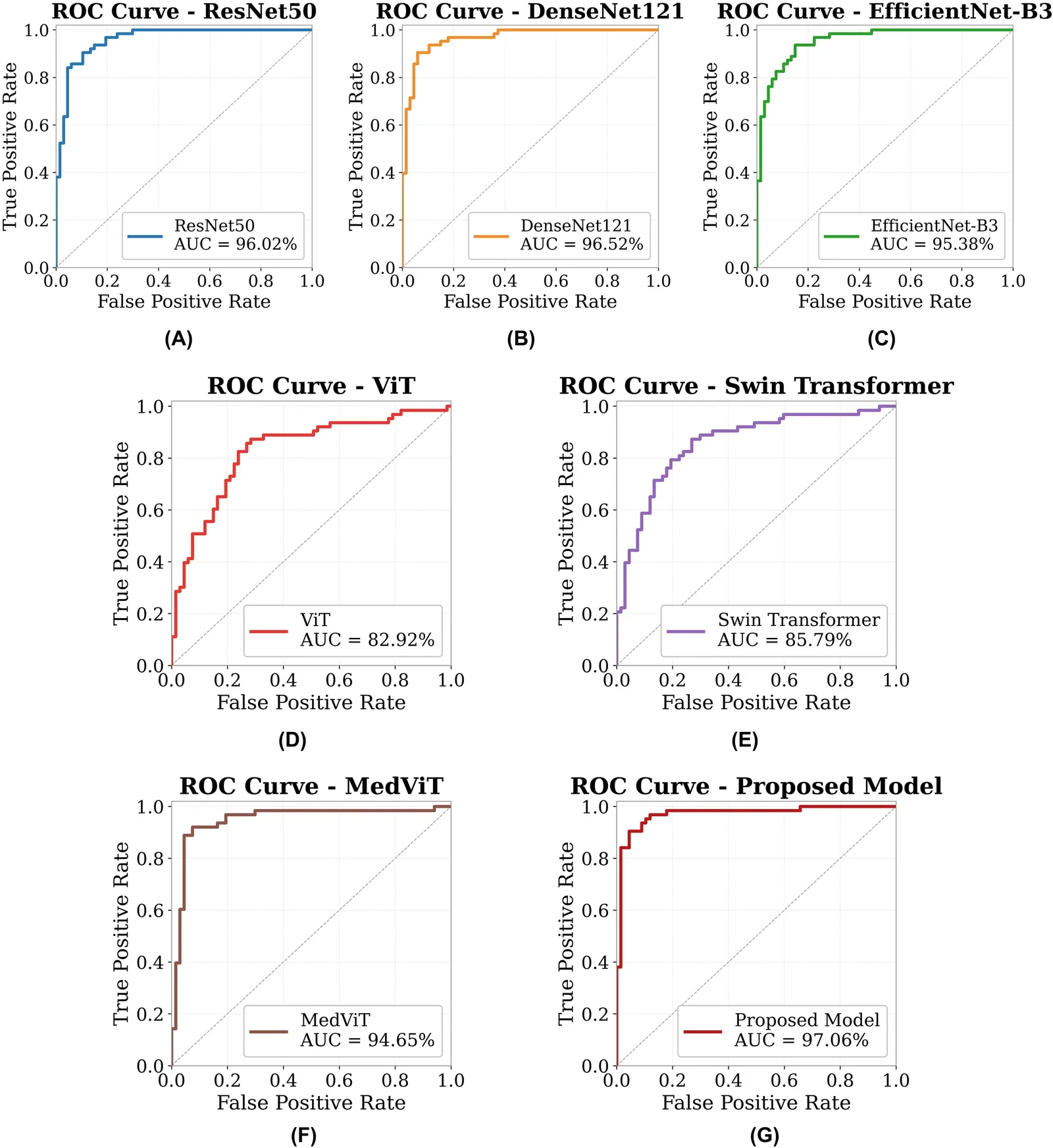
Receiver operating characteristic (ROC) curves of the evaluated models on the LIDC-IDRI test set. (A) ResNet50; (B) DenseNet121; (C) EfficientNet-B3; (D) Vision Transformer (ViT); (E) Swin Transformer; (F) MedViT; and (G) the proposed tri-view model. The area under the ROC curve (AUC) for each model is reported in the corresponding subplot.

The corresponding receiver operating characteristic (ROC) curves are shown in Fig 7.

The proposed tri-view model achieved an ACC of 93.08% (95% CI: 88.46–96.92%), an AUC of 97.06% (95% CI: 93.89–99.46%), and an F1-score of 92.68% (95% CI: 87.39–96.83%), with a sensitivity of 90.48% and a specificity of 95.52%. It obtained the highest ACC, AUC, and F1-score among all evaluated models. Among the single-view baselines, MedViT achieved the highest ACC (92.31%), F1-score (91.80%), and specificity (95.52%), DenseNet121 achieved the highest AUC (96.52%), and EfficientNet-B3 achieved the highest sensitivity (96.83%). Relative to the strongest single-view baseline for each metric, the proposed model improved ACC, AUC, and F1-score by 0.77, 0.54, and 0.88 percentage points, respectively, while matching the highest specificity. Bootstrap-derived 95% CIs for all evaluated models are provided in S2 Dataset.

### Ablation study

The ablation results are summarized in Table 3.

**Table 3:** Ablation study on the impact of key modules on model performance on the LIDC-IDRI dataset.

Model	Multi-view	LGA	VWF	ACC(%)	AUC(%)	F1(%)
**Baseline**	✗	✗	✗	92.31	94.65	91.80
**MV**	✓	✗	✗	90.77	95.78	90.32
**MV + VWF**	✓	✗	✓	90.77	96.14	90.32
**MV + LGA**	✓	✓	✗	91.54	**97.28**	91.34
**Full**	✓	✓	✓	**93.08**	97.06	**92.68**

^a^Baseline denotes the single-view model without multi-view input or fusion modules.

^b^MV: multi-view input; LGA: Lesion-Guided Attention; VWF: View-Weighted Fusion.

^c^All experiments used the same data partition, preprocessing pipeline, loss function, model-selection criterion, and evaluation protocol; ACC, AUC, and F1-score are reported as percentages.

^d^✓ indicates the module is used; ✗ indicates the module is removed.

The single-view Baseline achieved an ACC of 92.31%, an AUC of 94.65%, and an F1-score of 91.80%. Introducing tri-view input with direct concatenation (MV) increased the AUC to 95.78%, while the ACC and F1-score decreased to 90.77% and 90.32%, respectively.

Compared with direct concatenation in the MV configuration, replacing it with VWF increased the AUC from 95.78% to 96.14%, while the ACC and F1-score remained unchanged at 90.77% and 90.32%, respectively. The MV + LGA configuration improved the ACC, AUC, and F1-score to 91.54%, 97.28%, and 91.34%, respectively. The Full configuration achieved the highest ACC (93.08%) and F1-score (92.68%), whereas its AUC (97.06%) was slightly lower than that of MV + LGA (97.28%). Compared with the single-view Baseline, the Full configuration improved ACC, AUC, and F1-score by 0.77, 2.41, and 0.88 percentage points, respectively.

### Grad-CAM visualization results

**Fig 8:**
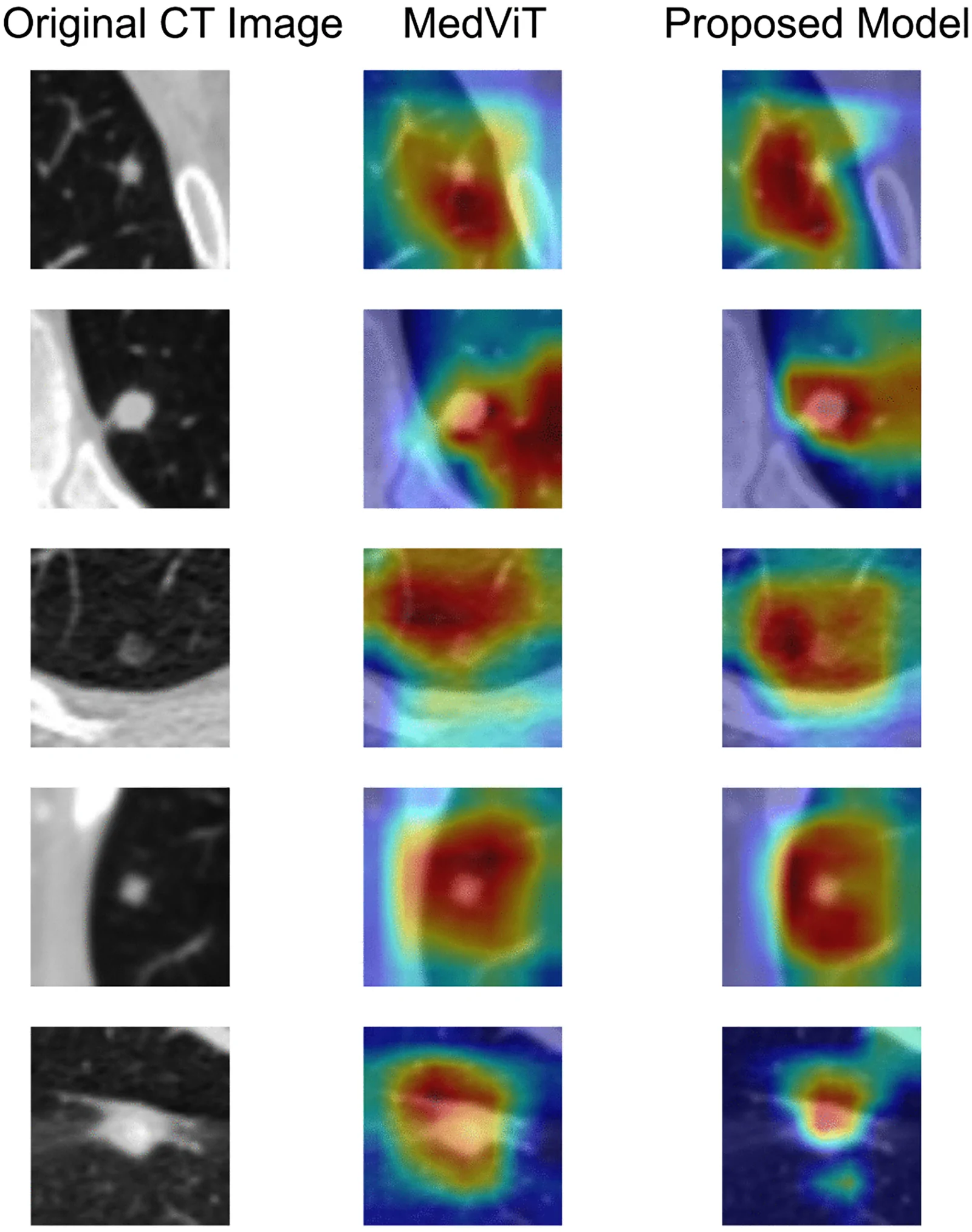
Grad-CAM comparison of the MedViT baseline and proposed multi-view model.

Selected Grad-CAM visualizations for test-set nodules are presented in Fig 8. The first column shows the original nodule images, while the second and third columns show the heatmaps generated by the MedViT baseline and the proposed multi-view model, respectively.

In the displayed cases, the MedViT baseline showed more diffuse activation patterns, with some responses extending into the surrounding lung tissue. The proposed model showed activations that were more concentrated within or adjacent to the nodule region. In several displayed cases, prominent responses were observed around portions of the nodule margins and internal regions.

## Discussion

This study evaluated a tri-view pulmonary nodule classification framework combining lesion-prior-guided soft residual feature modulation with sample-adaptive view-weighted fusion. On the held-out test set, the proposed multi-view model achieved an ACC of 93.08%, an AUC of 97.06%, and an F1-score of 92.68%, obtaining the highest values for these three metrics among the models included in the baseline comparison. In the ablation study, the Full configuration achieved the highest ACC and F1-score, whereas MV + LGA achieved a slightly higher AUC. Its specificity of 95.52% also matched the highest single-view result. These findings support the effectiveness of combining lesion-aware feature refinement with adaptive multi-view fusion for pulmonary nodule classification.

Tri-planar representation provides complementary spatial information but is not itself considered the methodological novelty of this study. Compared with the closely related framework of Chang et al. [13], the present model uses lesion masks as weak spatial priors for high-level soft residual feature refinement and learns sample-specific, Softmax-normalized weights for adaptive view fusion. Table 4 summarizes these conceptual differences.

**Table 4:** Conceptual comparison between the closest related multiview study and the present framework.

Aspect	Chang et al. (2024) [13]	Present study
**Tri-planar representation**	Uses axial, coronal, and sagittal CT views.	Uses the slice with the largest lesion-mask area in each plane.
**Primary methodological focus**	Multiview residual selective-kernel feature extraction and concatenation of homogeneity texture maps with CT intensity images.	Lesion-prior-guided soft residual feature modulation and sample-adaptive view-weighted fusion.
**Use of lesion masks**	The published framework description does not report lesion-mask-derived soft residual modulation.	Lesion masks are used as weak spatial priors at the high-level feature stage; they guide attention but are not used as hard cropping boundaries or direct classification inputs.
**Perinodular context**	Retention of perinodular context is not explicitly presented as a design objective.	Limited perinodular context is retained through soft modulation and residual feature preservation rather than hard mask-based exclusion.
**Cross-view fusion**	The reported design emphasizes multiview representation and CT/texture-map concatenation; sample-specific Softmax-normalized view weighting is not reported.	Each view receives a sample-specific score; Softmax produces non-negative weights that sum to one for weighted feature aggregation.
**Evaluation design**	Ten-fold cross-validation on LIDC-IDRI.	Stratified nodule-level training/validation/test split with baseline and module ablation experiments.

^a^This table provides a conceptual comparison based on the methodological descriptions reported in the cited studies. It is not intended as a head-to-head performance comparison because the data partitioning, sample selection, preprocessing, and evaluation protocols differ.

Because case selection, label definitions, exclusion criteria, data partitioning, preprocessing pipelines, input representations, and evaluation protocols differ across studies, cross-study performance values were not interpreted as direct evidence of superiority. The quantitative conclusions of the present study therefore rely primarily on the baseline comparison and ablation experiments conducted using the same nodule-level data partition and evaluation protocol.

The ablation results showed that tri-view input with direct concatenation increased AUC but reduced ACC and F1-score relative to the single-view Baseline. Replacing direct concatenation with VWF further increased AUC, while ACC and F1-score remained unchanged. By contrast, adding LGA to MV improved all three reported metrics and produced the highest AUC among the evaluated configurations. The Full configuration achieved the highest ACC and F1-score, although its AUC was slightly lower than that of MV + LGA. These results indicate that the modules affected AUC and threshold-dependent metrics differently. AUC evaluates the model’s ability to rank malignant nodules above benign nodules across different decision thresholds, whereas ACC and F1-score depend on the single decision threshold selected from the validation set. Direct concatenation and VWF improved AUC without corresponding improvements in ACC and F1-score, while LGA improved all three metrics. Accordingly, the Full model achieved the highest ACC and F1-score at the selected threshold, whereas MV + LGA achieved the highest AUC.

Selected Grad-CAM visualizations provided qualitative information regarding the spatial patterns associated with model predictions. In the displayed cases, the proposed model showed activation patterns that were less diffuse in the surrounding lung tissue and more concentrated within or adjacent to the nodule region than those of the MedViT baseline. These observations are useful for qualitative inspection of whether model predictions are associated with lesion-relevant regions. However, Grad-CAM heatmaps should not be interpreted as quantitative localization measurements, clinical evidence, or causal explanations of model decisions.

From a potential clinical-use perspective, the proposed framework could serve as a computer-aided second-reader tool after a pulmonary nodule has been identified and a lesion mask is available. The consensus masks used in this study were derived from existing LIDC-IDRI annotations and do not imply that four radiologists would be required to delineate each lesion in future clinical use. In practice, the required lesion mask could potentially be obtained from an existing clinical contour or an automated segmentation method, although these alternatives were not evaluated in the present study. The model could then provide a malignancy probability based on complementary tri-view information to support radiologist assessment rather than replace the radiologist’s diagnostic judgment. However, prospective clinical evaluation, including its effect on reader performance and workflow efficiency, is required before such use can be established.

Several limitations should be acknowledged. First, all experiments were conducted on a single LIDC-IDRI cohort, and no independent external validation was performed. The LGA module also relies on annotation-derived lesion masks as weak spatial priors. Application to external data would require manual or automatically generated masks, and robustness to imperfect masks remains to be assessed. In addition, data partitioning was performed at the nodule level rather than the patient level. Consequently, distinct nodules from the same patient could occur in different subsets, introducing residual patient-level correlation and potentially yielding less conservative performance estimates than a fully patient-independent evaluation. Although no individual nodule or its derived tri-view images and masks crossed subsets, future studies should evaluate the framework using patient-level partitions and independent external cohorts.

Second, the benign–malignant labels were defined from the median radiologist-assigned malignancy scores rather than pathological confirmation. Excluding nodules with a median score of 3 reduced label ambiguity but restricted the evaluation to relatively less ambiguous nodules. Third, selecting only one two-dimensional slice with the largest lesion-mask area in each view does not fully capture continuous volumetric context. Future work should assess the framework on independent multi-center cohorts, evaluate its robustness under heterogeneous acquisition conditions and imperfect segmentation masks, incorporate relevant clinical variables, and investigate efficient volumetric modeling strategies.

## Conclusion

We developed a tri-view pulmonary nodule classification framework that integrates lesion-prior-guided soft residual feature modulation with sample-adaptive view-weighted fusion to exploit complementary information from axial, coronal, and sagittal views. On the held-out LIDC-IDRI test set, the proposed framework achieved an ACC of 93.08%, an AUC of 97.06%, and an F1-score of 92.68%. The ablation results showed that combining LGA and VWF provided the best overall performance balance among the evaluated configurations. In the displayed test cases, Grad-CAM visualizations showed that model activations were concentrated mainly within or adjacent to lesion-relevant regions.

Because the study was conducted using a single public dataset and annotation-derived lesion masks, independent multi-center validation and further evaluation with imperfect or automatically generated masks are needed before broader application.

## Supporting information

S1 Dataset Nodule-level data-partition manifest. This file contains the nodule identifiers, ground-truth labels, and training, validation, or test-set assignment for the 869 eligible nodules. (ZIP)

S2 Dataset Per-nodule prediction results, validation-derived classification thresholds, and bootstrap confidence intervals. This file contains the ground-truth labels, per-nodule prediction probabilities, validation-derived decision thresholds, and bootstrap-derived 95% confidence intervals for the evaluated models.
(ZIP)

S1 Raw Images Source images underlying the image-based figures reported in the manuscript. This study did not involve blot or gel experiments. The file includes computer-generated methodological schematics, CT images, radiologist-provided annotations, consensus masks, tri-view model-input images, ROC curves, and Grad-CAM visualizations.
(PDF)
