# Supplementary material for: Multi-view attention-based deep learning for benign–malignant classification of pulmonary nodules
Source: PLoS One. 2026 Sep 15;21(9):e0357963. doi: 10.1371/journal.pone.0357963 (PMC13577567; doi:10.1371/journal.pone.0357963)
Supplement: Supplementary file 1 [file pone.0357963.s003.pdf]

# S1_raw_images

This manuscript does not report western blots, electrophoretic gels, or any other blot/gel experiments. Therefore, no blot or gel images, experimental lanes, molecular-weight markers, or exposure information exist for this study.

This file is provided in response to the journal’s technical request. It contains the source images underlying the image-based figures in the manuscript, including computer-generated methodological schematics, CT images, radiologist-provided annotations, consensus masks, tri-view model-input images, ROC plots, and Grad-CAM visualizations derived from the publicly available LIDC-IDRI dataset.

# Fig 1

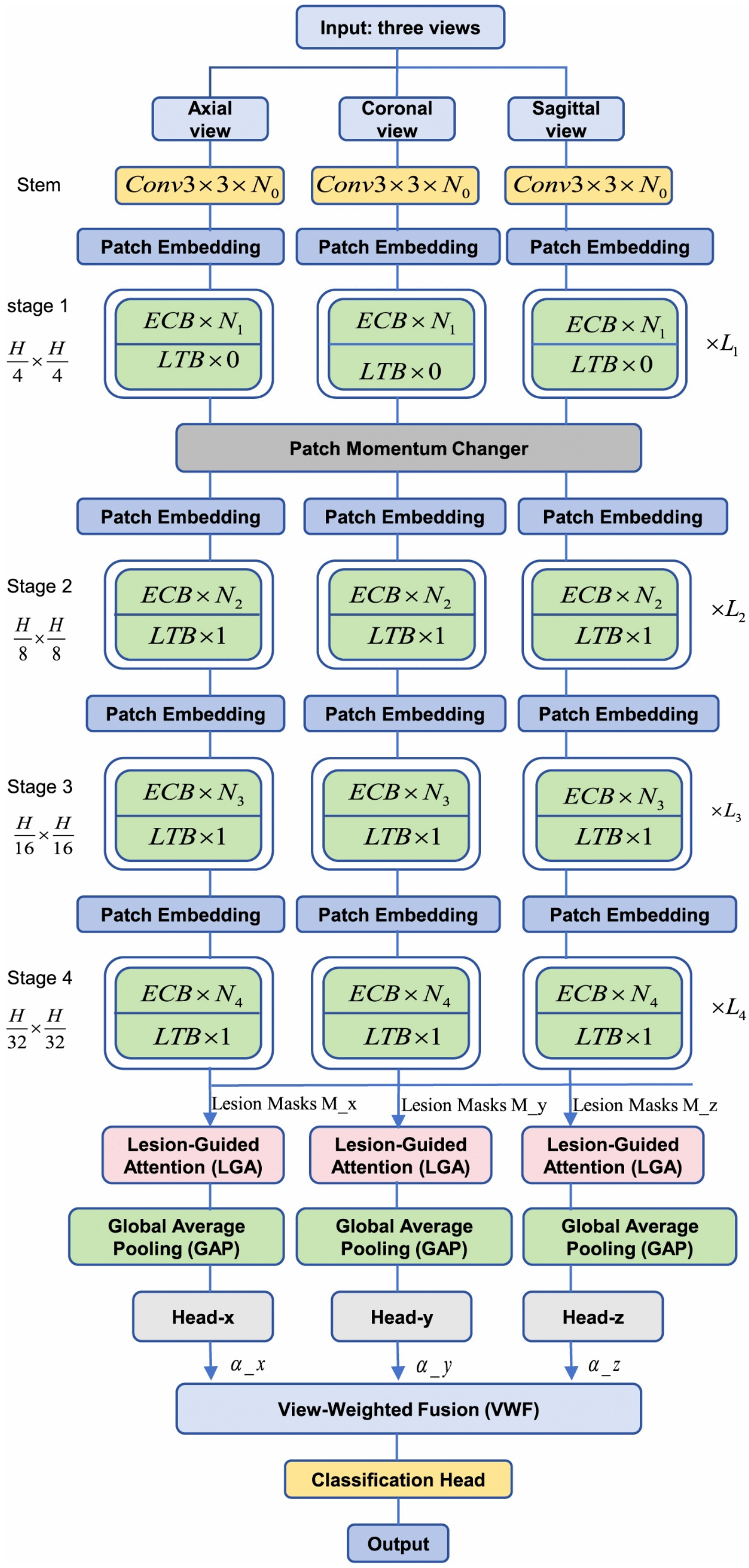


## Source information

Corresponding manuscript figure: Fig 1
Figure type: computer-generated methodological schematic
Raw experimental image: Not applicable
Source file: Fig1.tif
No blot/gel lanes, molecular-weight markers, or exposure settings are applicable.

# Fig 2

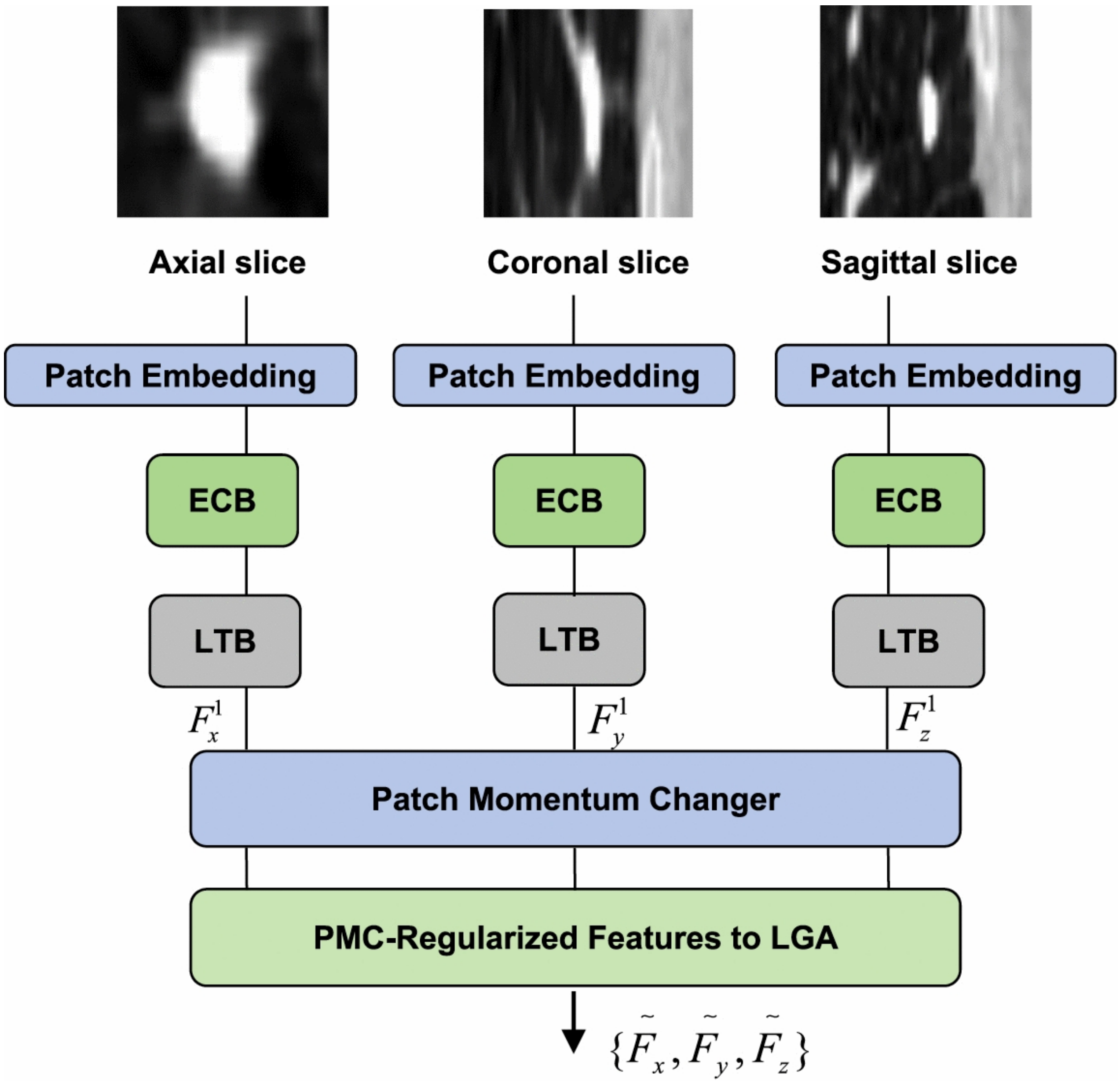


## Source information

Corresponding manuscript figure: Fig 2
Figure type: computer-generated methodological schematic
Raw experimental image: Not applicable
Source file: Fig2.tif
No blot/gel lanes, molecular-weight markers, or exposure settings are applicable.

# Fig 3

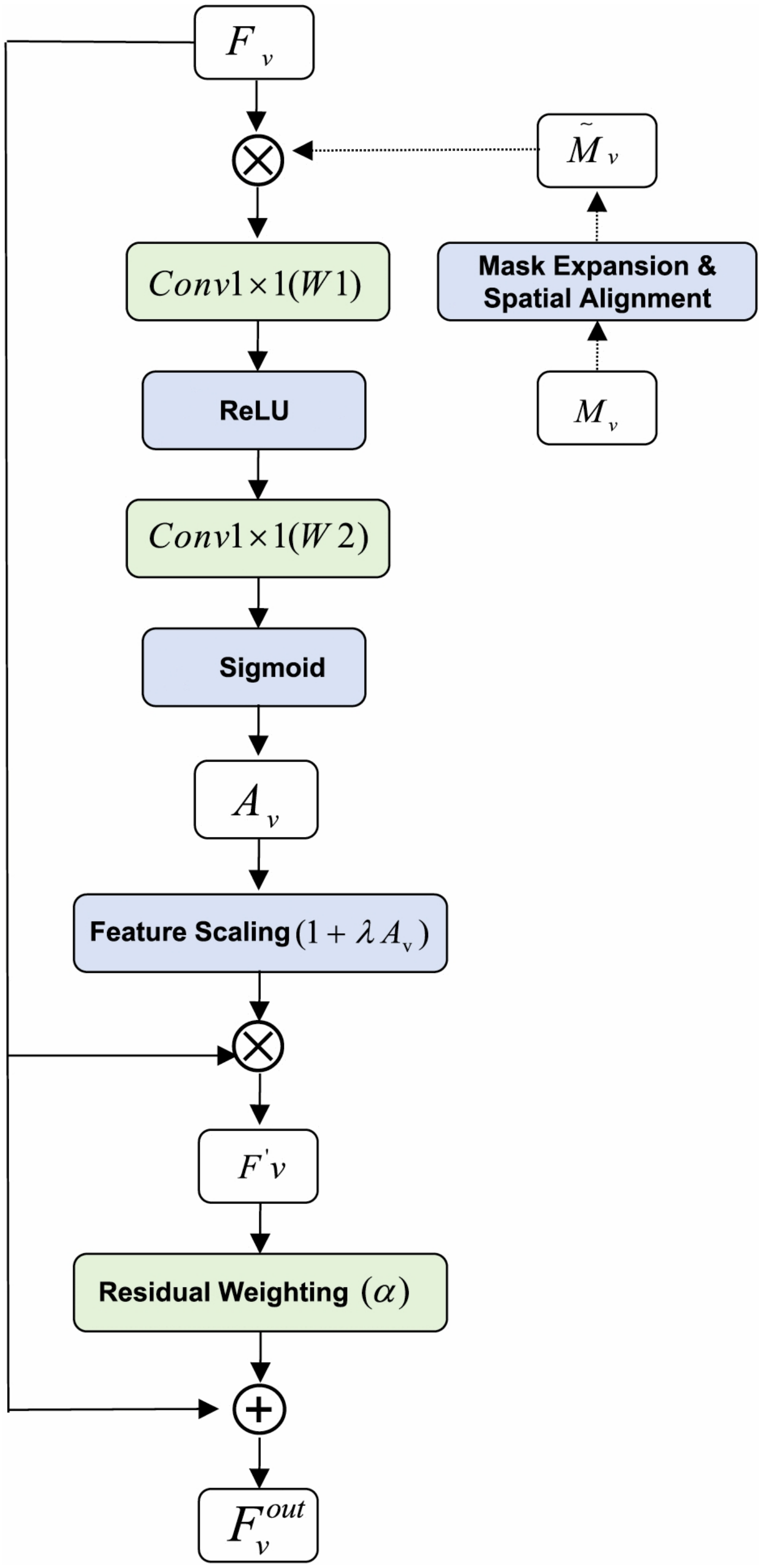


## Source information

Corresponding manuscript figure: Fig 3
Figure type: computer-generated methodological schematic
Raw experimental image: Not applicable
Source file: Fig3.tif
No blot/gel lanes, molecular-weight markers, or exposure settings are applicable.

# Fig 4

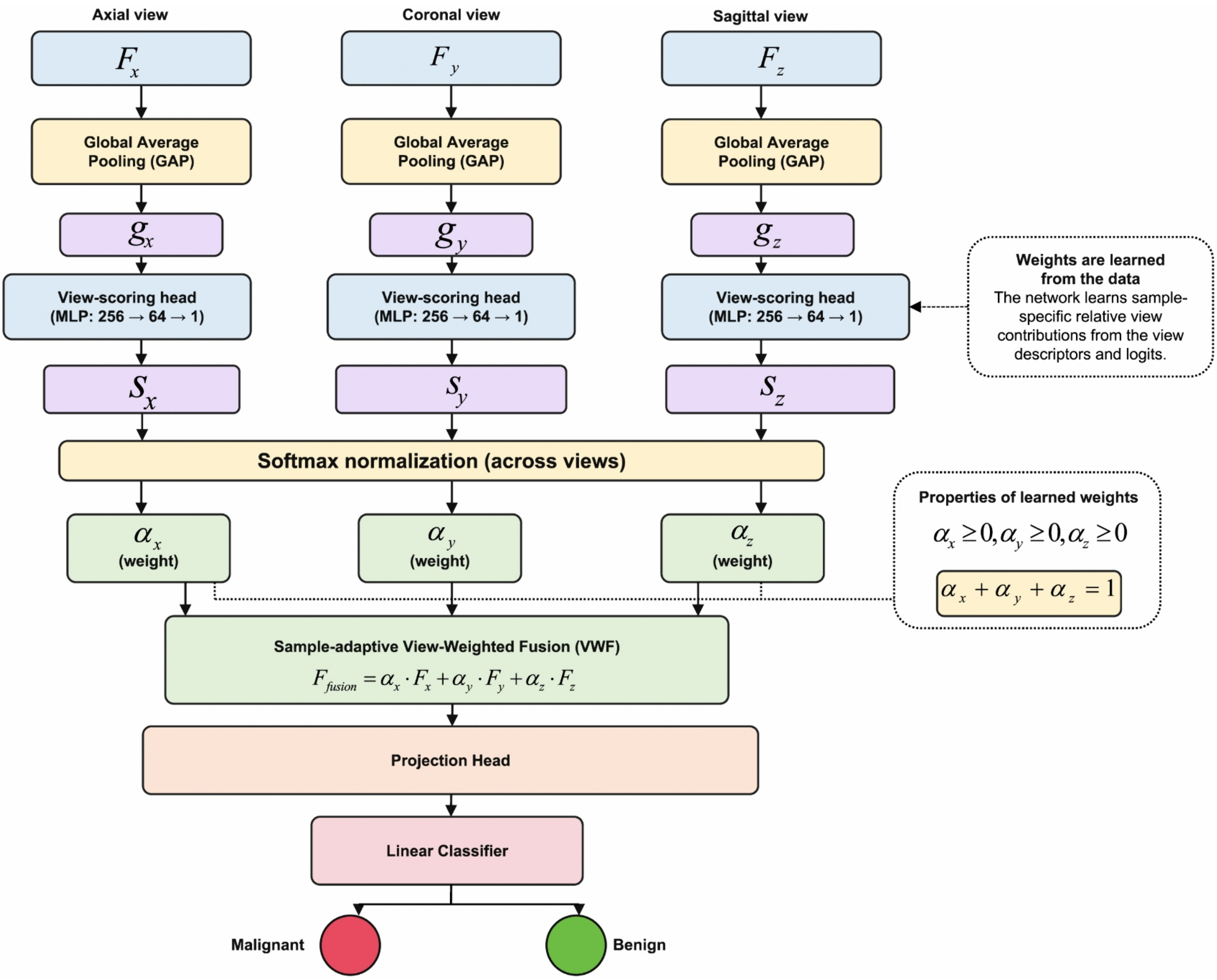


## Source information

Corresponding manuscript figure: Fig 4
Figure type: computer-generated methodological schematic
Raw experimental image: Not applicable
Source file: Fig4.tif
No blot/gel lanes, molecular-weight markers, or exposure settings are applicable.

# Fig 5

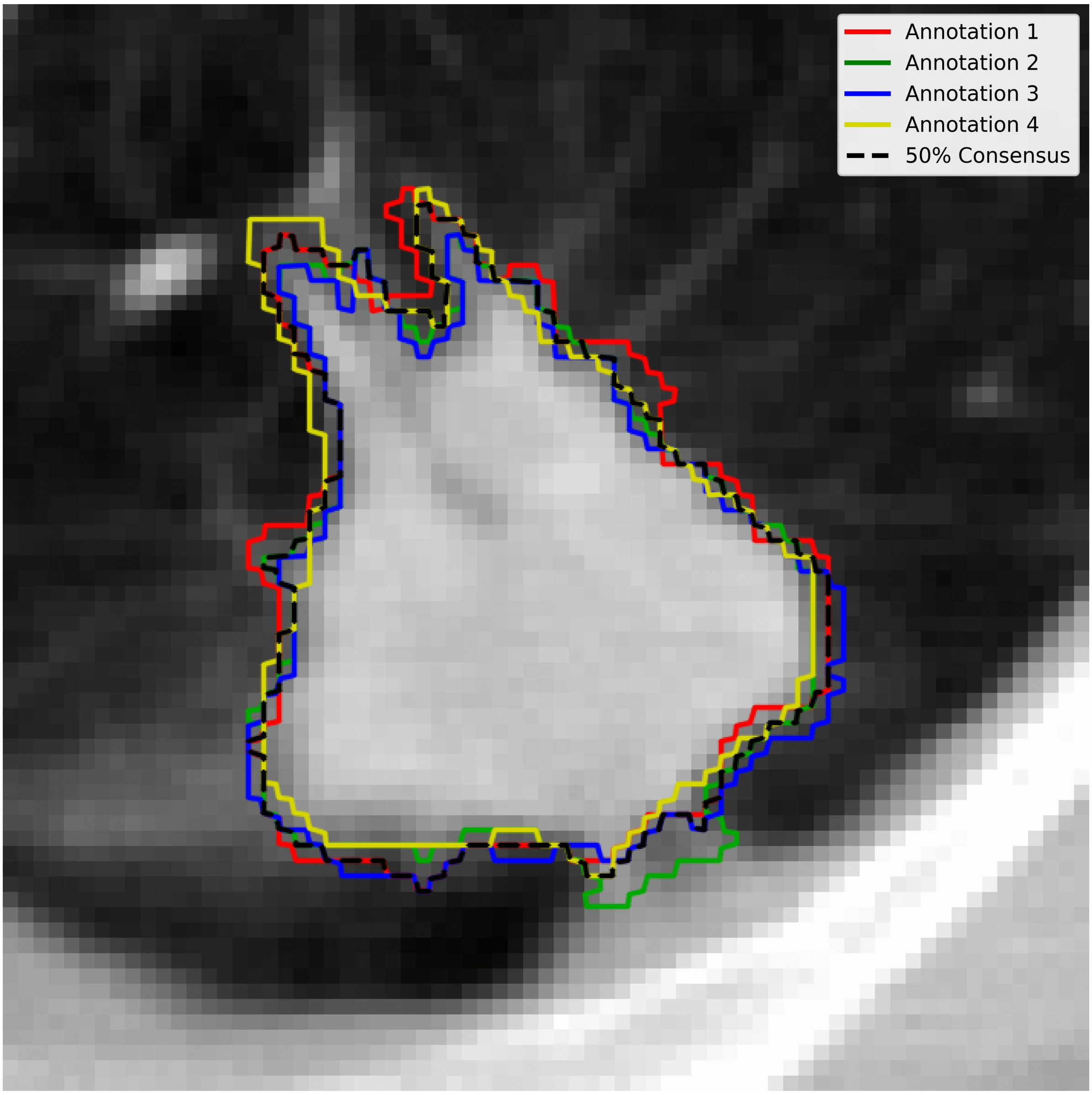


## Source information

Corresponding manuscript figure: Fig 5
Image type: Final assembled CT annotation figure
Source dataset: LIDC-IDRI / TCIA
Sample ID: LIDC-IDRI-0001_718b6593_N01
Patient/Case ID: LIDC-IDRI-0001
Nodule ID: N01
Ground-truth label: Malignant
Series UID:
1.3.6.1.4.1.14519.5.2.1.6279.6001.179049373636438705059720603192
Selected axial slice: Slice with the largest 50% consensus-mask area

# Fig 5. Underlying source images

Original axial CT slice

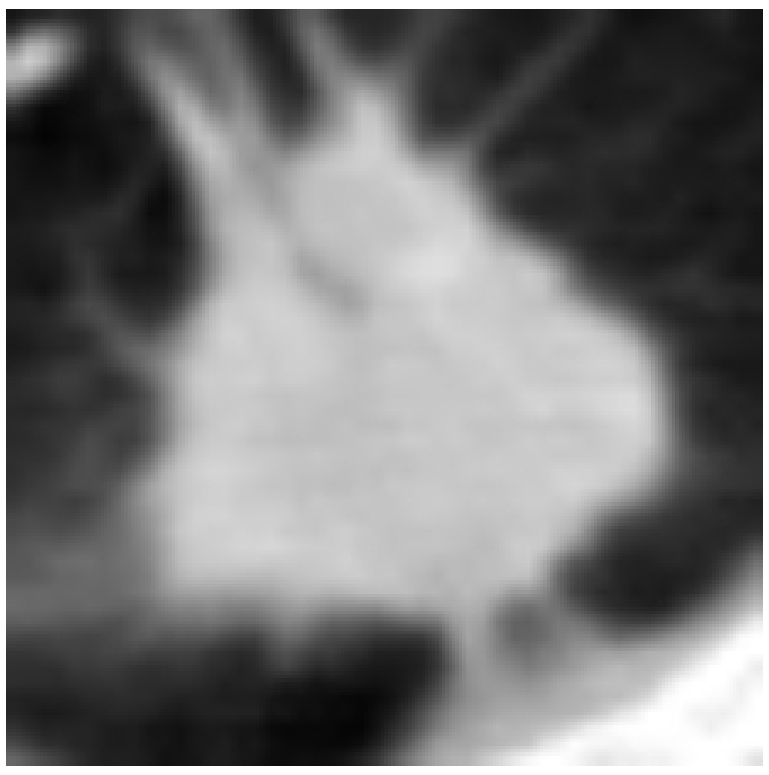

50% consensus mask

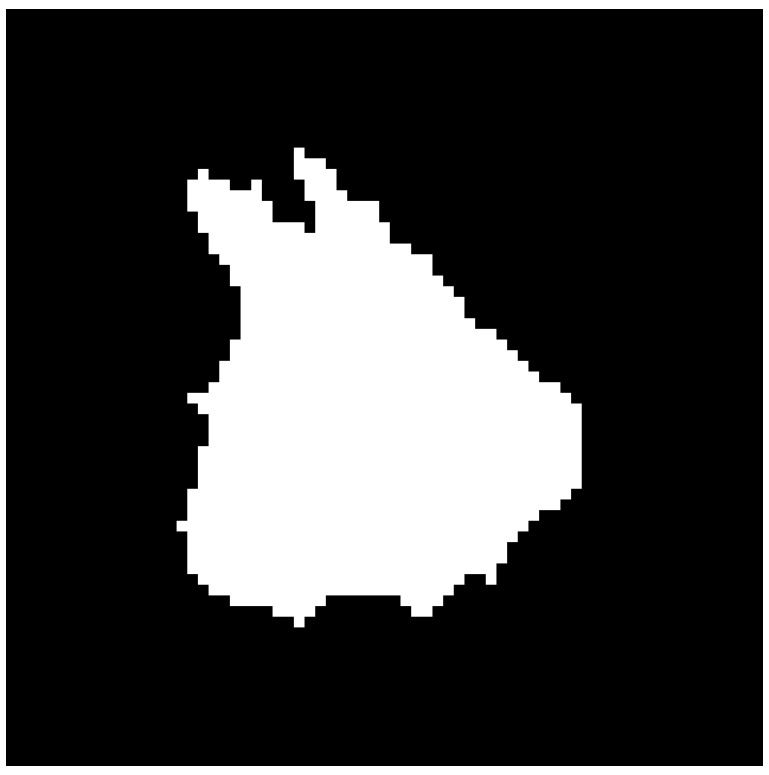

CT + Annotation 1 contour

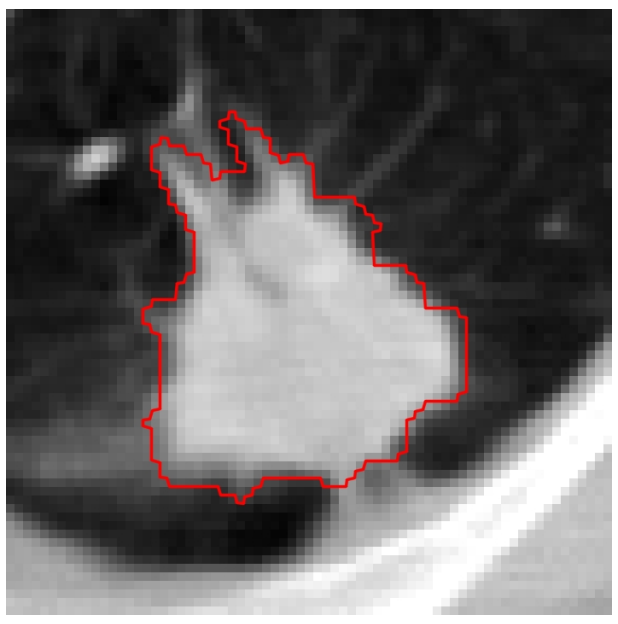

CT + Annotation 2 contour

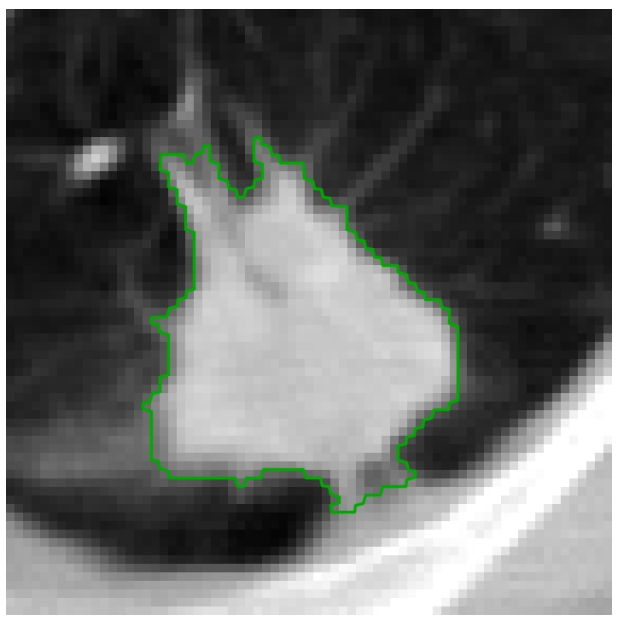

CT + Annotation 3 contour

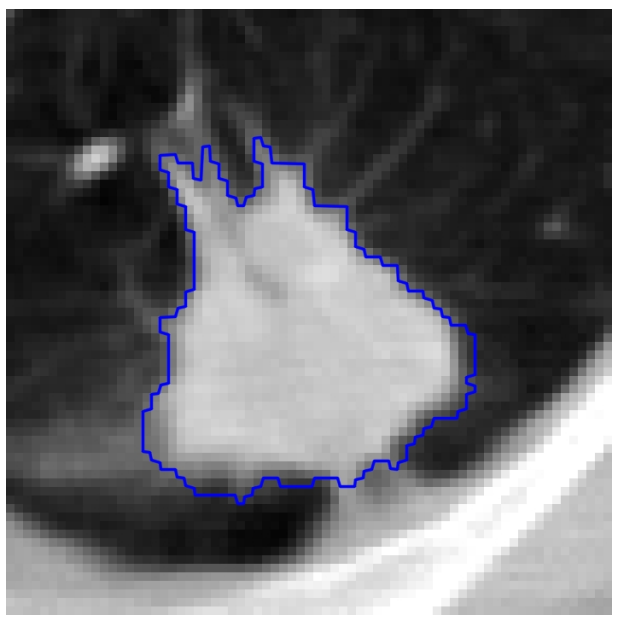

CT + Annotation 4 contour

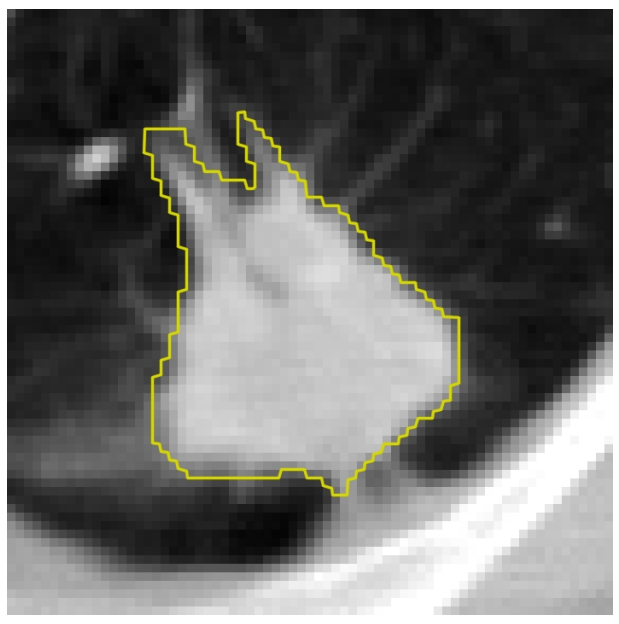

Final overlay

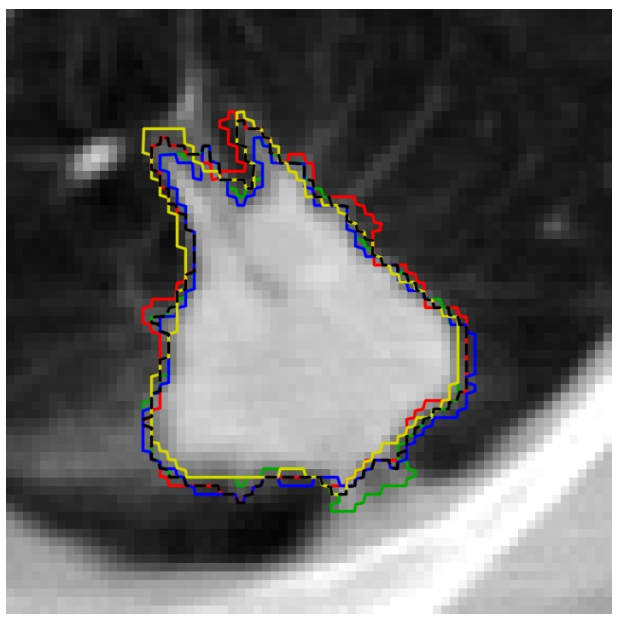

## Source information

Corresponding manuscript figure: Fig 5
Source dataset: LIDC-IDRI / TCIA
Sample ID: LIDC-IDRI-0001_718b6593_N01
Patient/Case ID: LIDC-IDRI-0001
Nodule ID: N01
Ground-truth label: Malignant
Series UID:
1.3.6.1.4.1.14519.5.2.1.6279.6001.179049373636438705059720603192
View and slice selection: Axial slice with the largest 50% consensus-mask area
Processing: Original DICOM intensities were converted to HU, clipped to
[-1000, 400], and linearly normalized. The image was cropped around the
annotated nodule and enlarged using nearest-neighbor interpolation for display.
Purpose: Source images underlying the four radiologist contours and the
50% consensus region shown in Fig 5.

# Fig 6

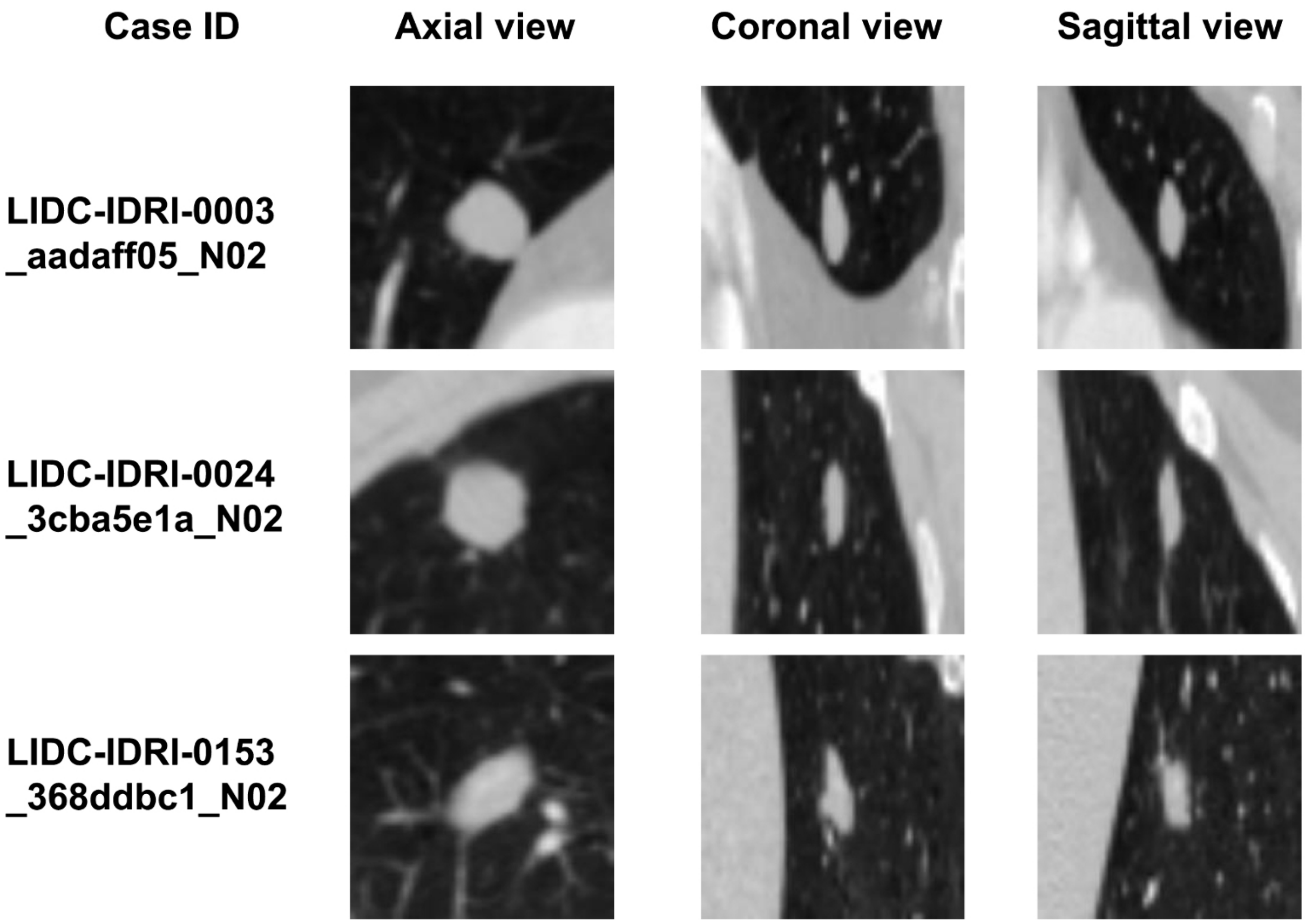


## Source information

Corresponding manuscript figure: Fig 6
Image type: Final assembled tri-view CT panel
Source dataset: LIDC-IDRI / TCIA
Unassembled axial, coronal, and sagittal model-input images for the three displayed nodules are provided on the following pages.

# Fig 6. Case 1 unassembled tri-view source images

Axial view source image

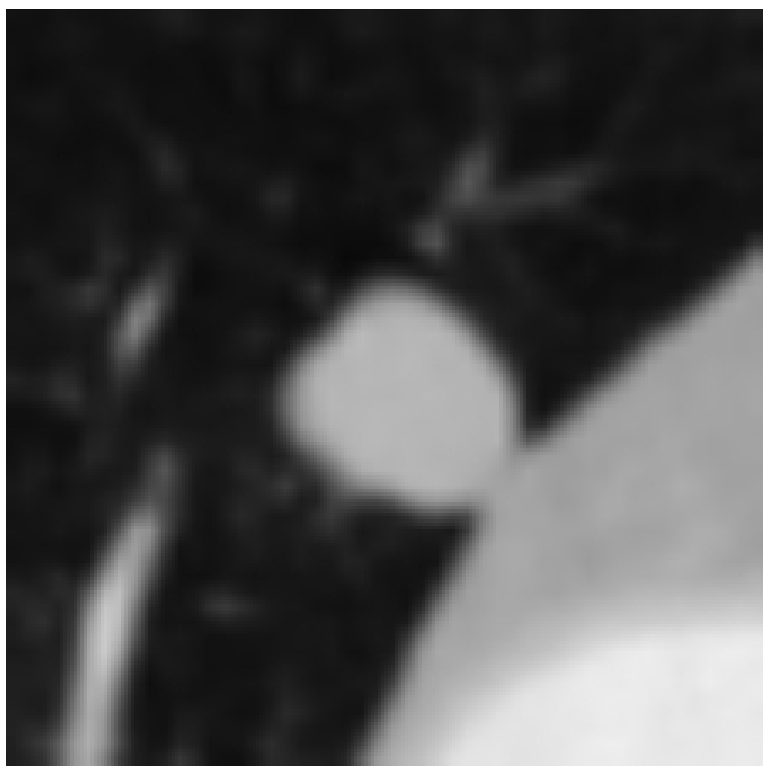

Coronal view source image

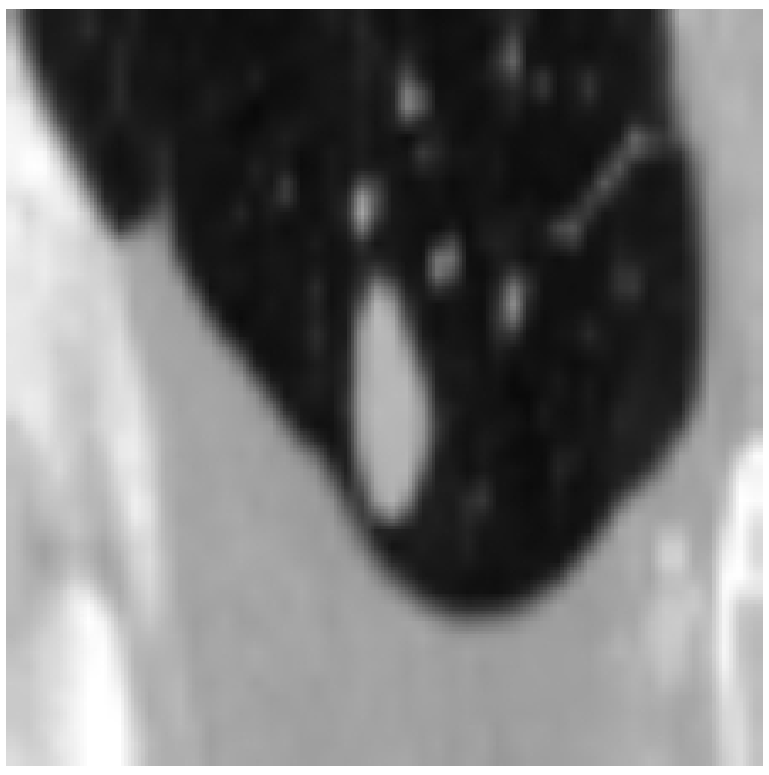

Sagittal view source image

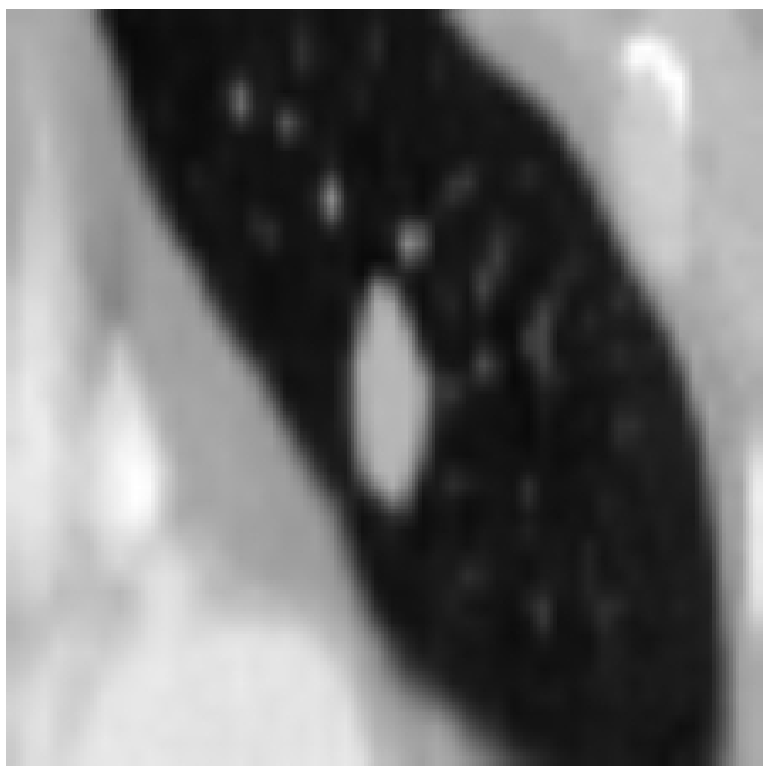

## Source information

Corresponding manuscript figure: Fig 6, Row 1
Sample ID: LIDC-IDRI-0003_aadaff05_N02
Patient/Case ID: LIDC-IDRI-0003
Nodule ID/index: N02
Ground-truth label: Malignant
Image type: Preprocessed and unassembled model-input images used to construct Fig 6
Patch size: 50 × 50 × 50 voxels
Displayed resolution: 224 × 224 pixels
Processing: HU clipping [-1000, 400], linear normalization, representative-slice selection, and resizing to 224 × 224. No random augmentation was applied during figure generation.

# Fig 6. Case 2 unassembled tri-view source images

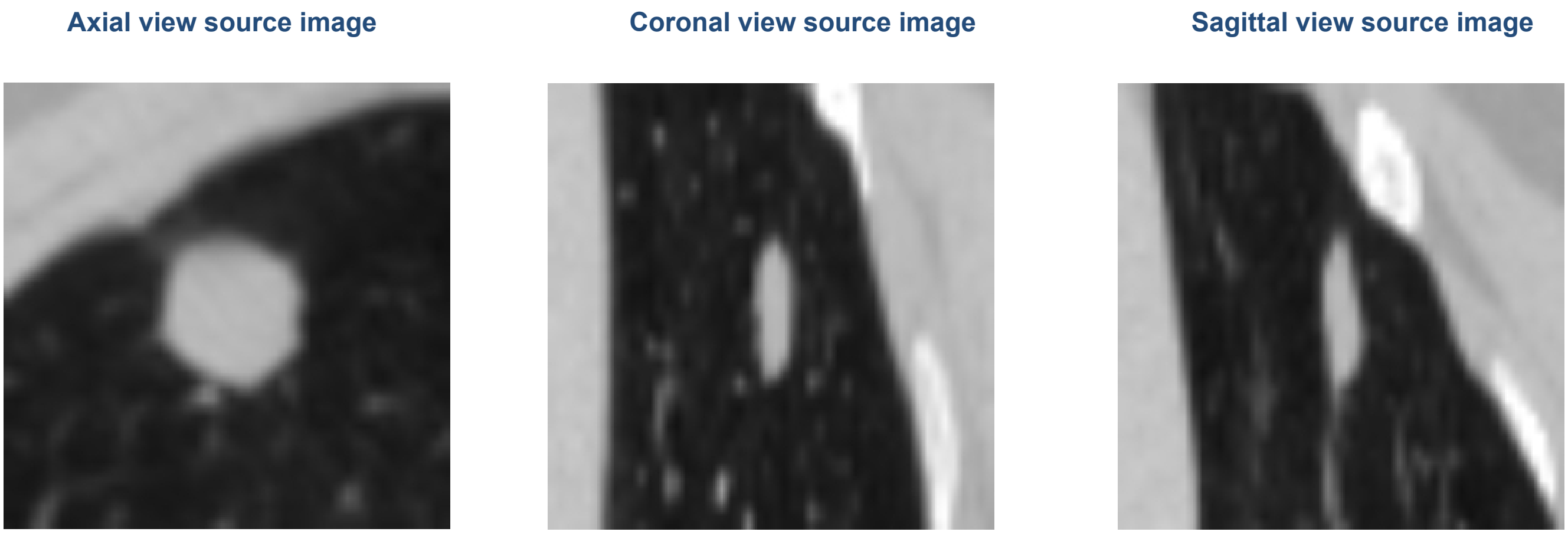


## Source information

Corresponding manuscript figure: Fig 6, Row 2
Sample ID: LIDC-IDRI-0024_3cba5e1a_N02
Patient/Case ID: LIDC-IDRI-0024
Nodule ID/index: N02
Ground-truth label: Malignant
Image type: Preprocessed and unassembled model-input images used to construct Fig 6
Patch size: 50 × 50 × 50 voxels
Displayed resolution: 224 × 224 pixels
Processing: HU clipping [-1000, 400], linear normalization, representative-slice selection, and resizing to 224 × 224. No random augmentation was applied during figure generation.

# Fig 6. Case 3 unassembled tri-view source images

Axial view source image

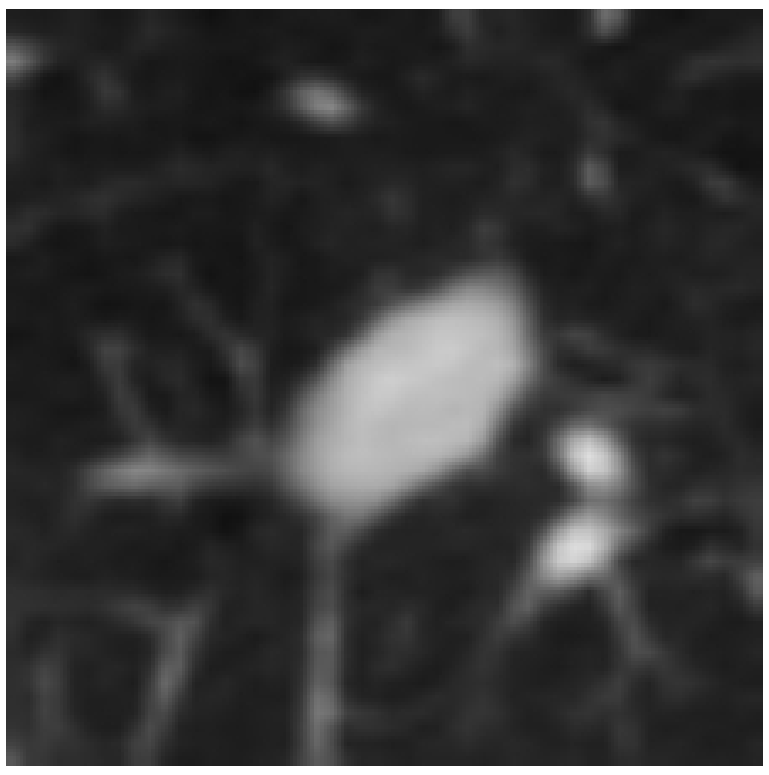

Coronal view source image

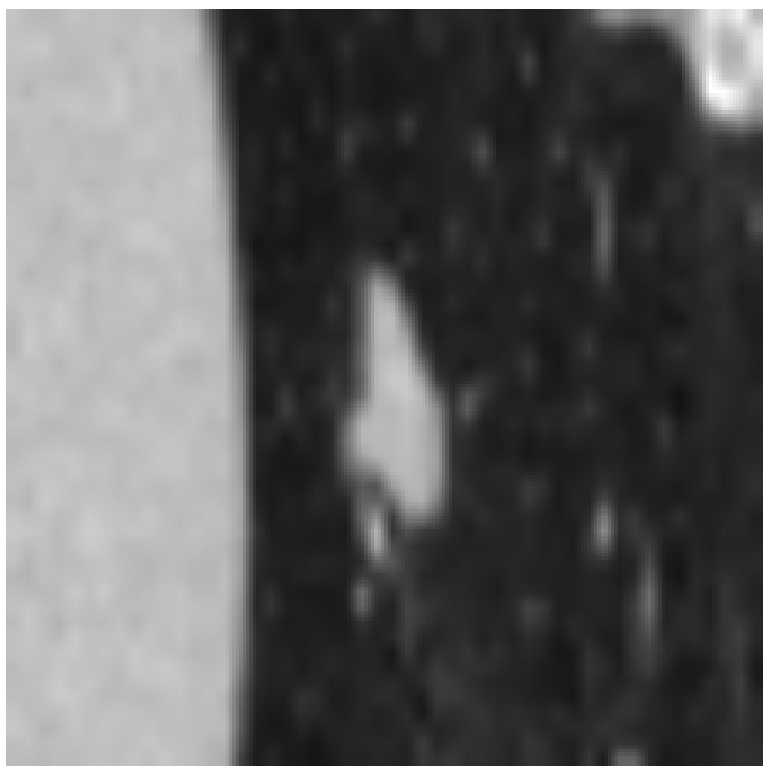

Sagittal view source image

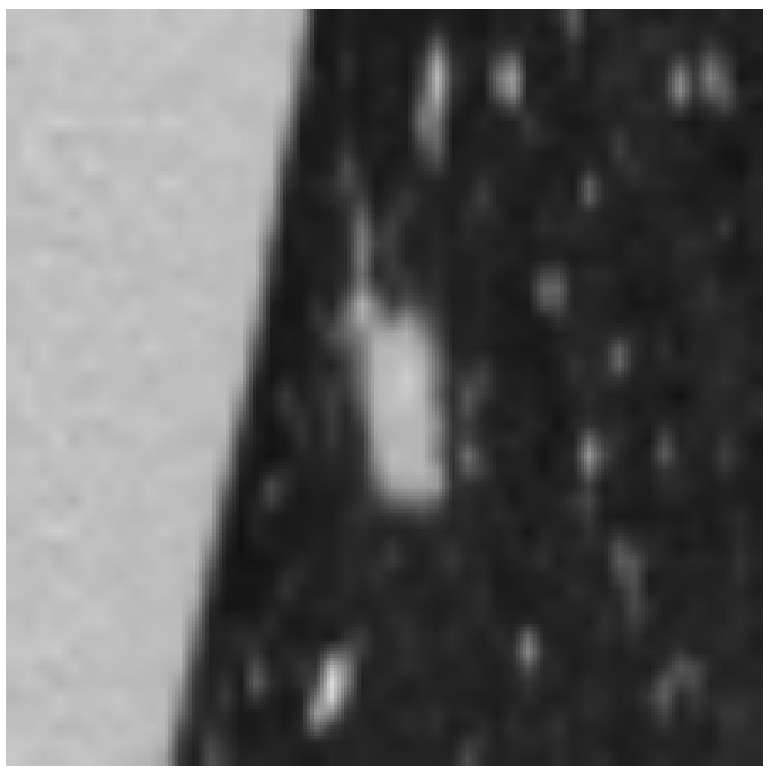

## Source information

Corresponding manuscript figure: Fig 6, Row 3
Sample ID: LIDC-IDRI-0153_368ddbc1_N02
Patient/Case ID: LIDC-IDRI-0153
Nodule ID/index: N02
Ground-truth label: Malignant
Image type: Preprocessed and unassembled model-input images used to construct Fig 6
Patch size: 50 × 50 × 50 voxels
Displayed resolution: 224 × 224 pixels
Processing: HU clipping [-1000, 400], linear normalization, representative-slice selection, and resizing to 224 × 224. No random augmentation was applied during figure generation.

# Fig 7

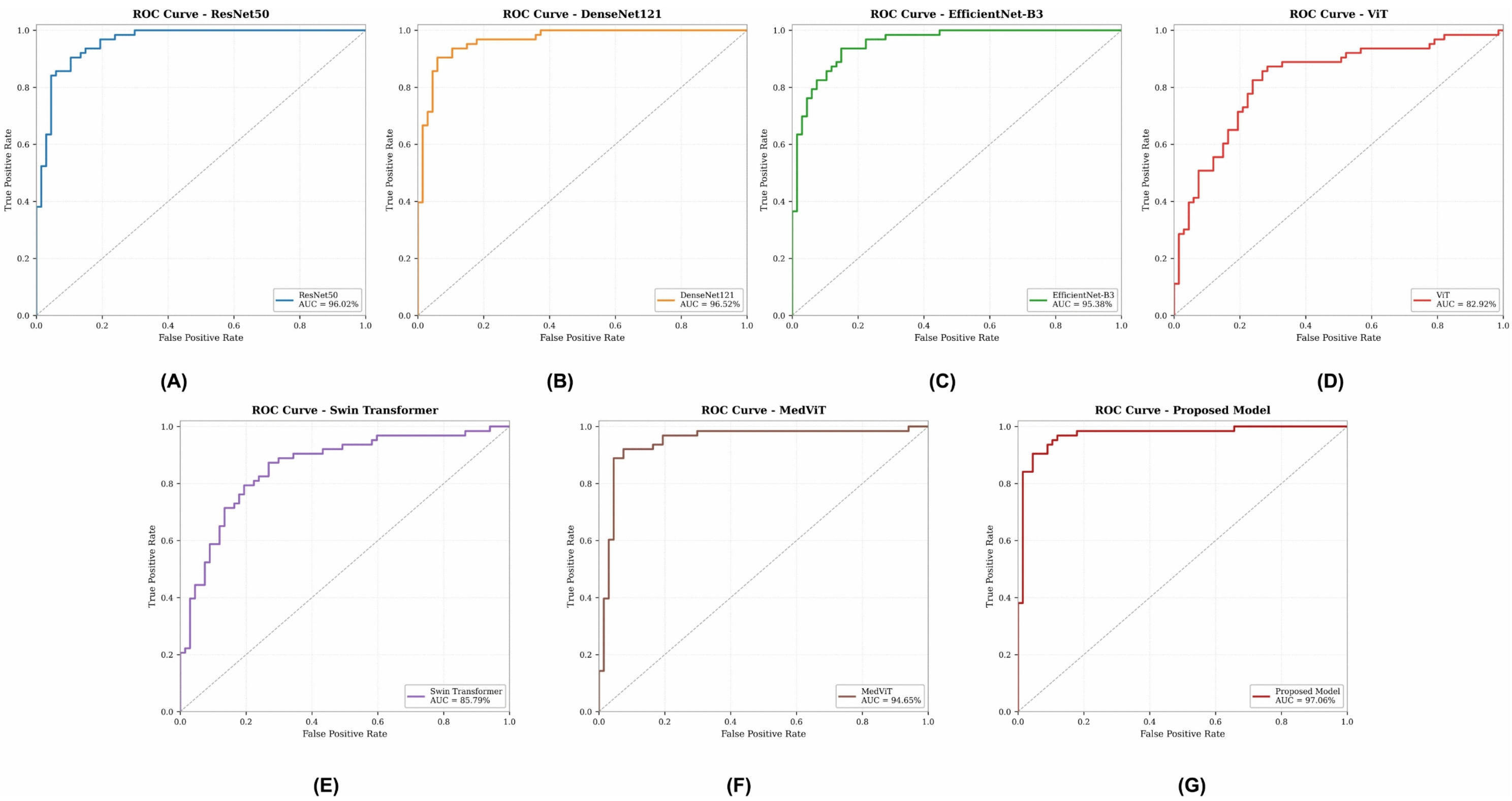


## Source information

Underlying data: The data underlying the ROC curves are provided in S2 Dataset, “Per-nodule prediction results and validation-derived classification thresholds,” including ground-truth labels, per-nodule prediction probabilities, and validation-derived decision thresholds for the evaluated baseline, ablation, and full-model configurations.

## Fig 8

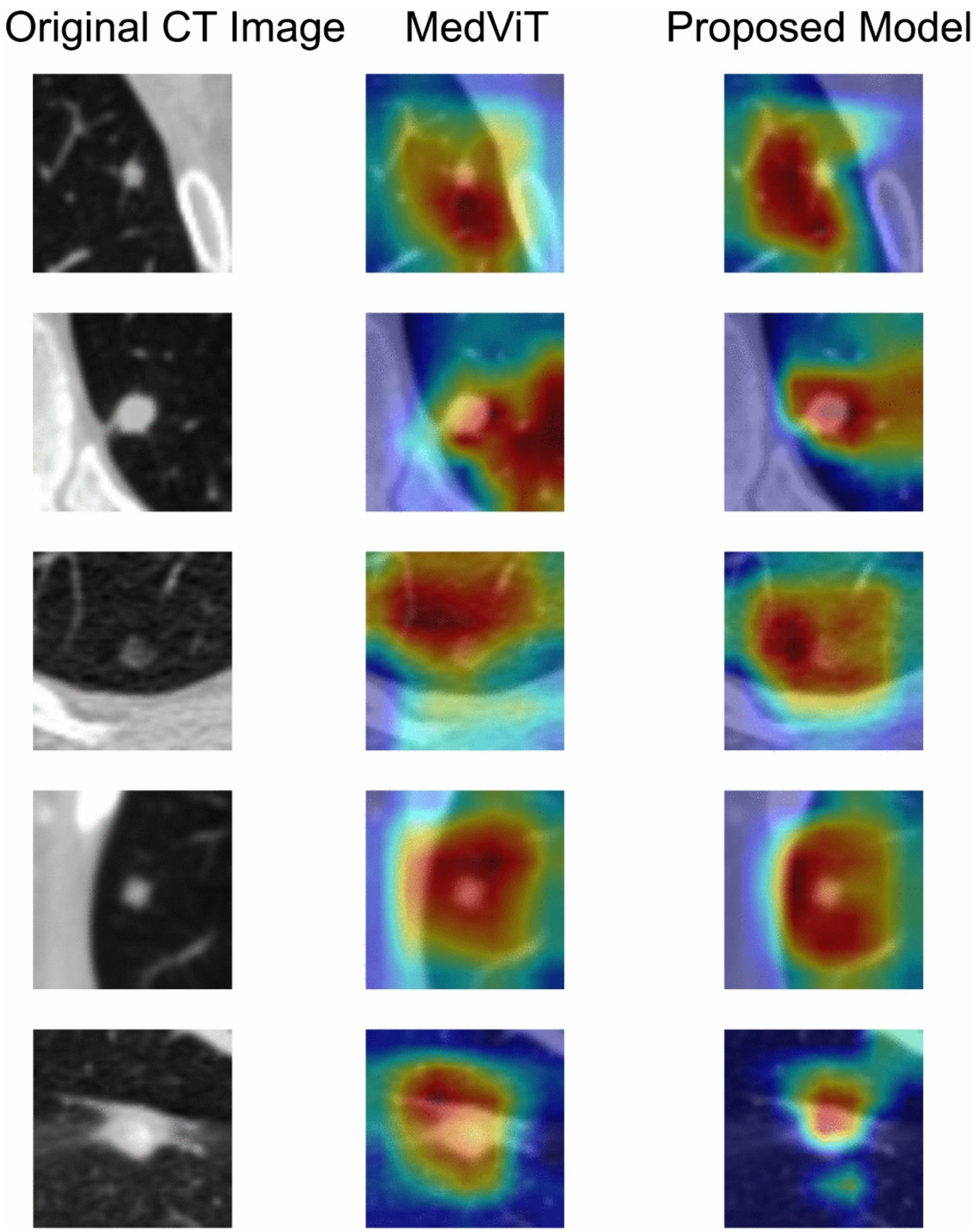


### Source information

Corresponding manuscript figure: Fig 8
Image type: Final assembled Grad-CAM comparison panel
Source images: Preprocessed axial CT inputs and unassembled Grad-CAM overlays
from the single-view MedViT baseline and the full proposed model.
The source images for the five displayed nodules are provided on pages 14–18.

# Fig 8. Case 1 unassembled Grad-CAM source images

Original CT input

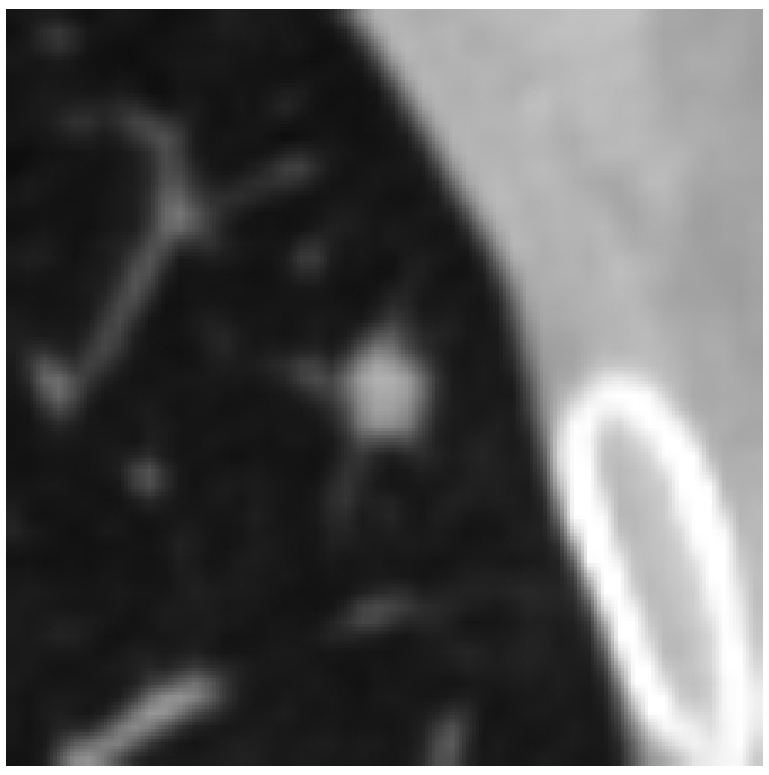

MedViT Grad-CAM

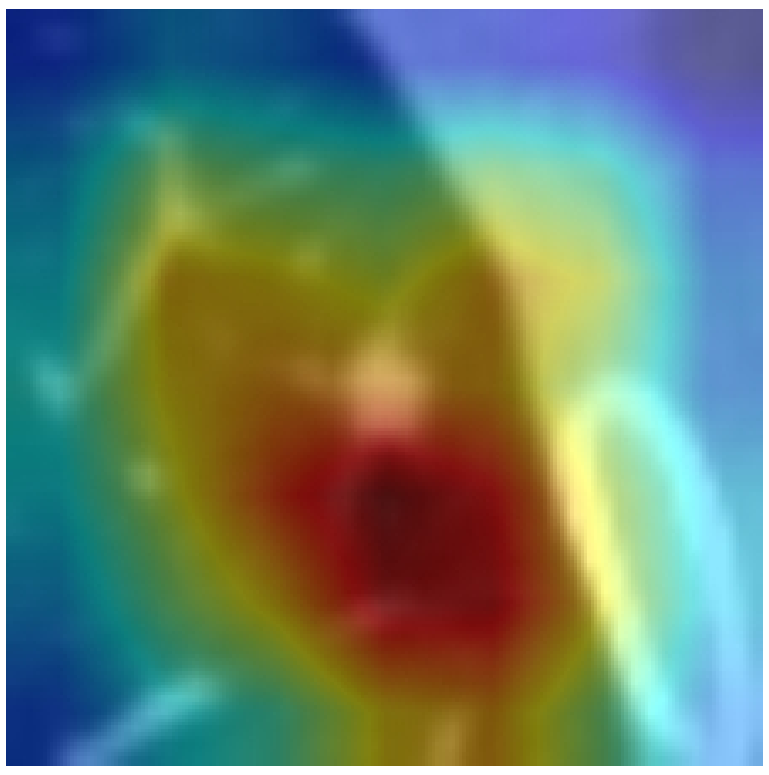

Proposed Model Grad-CAM

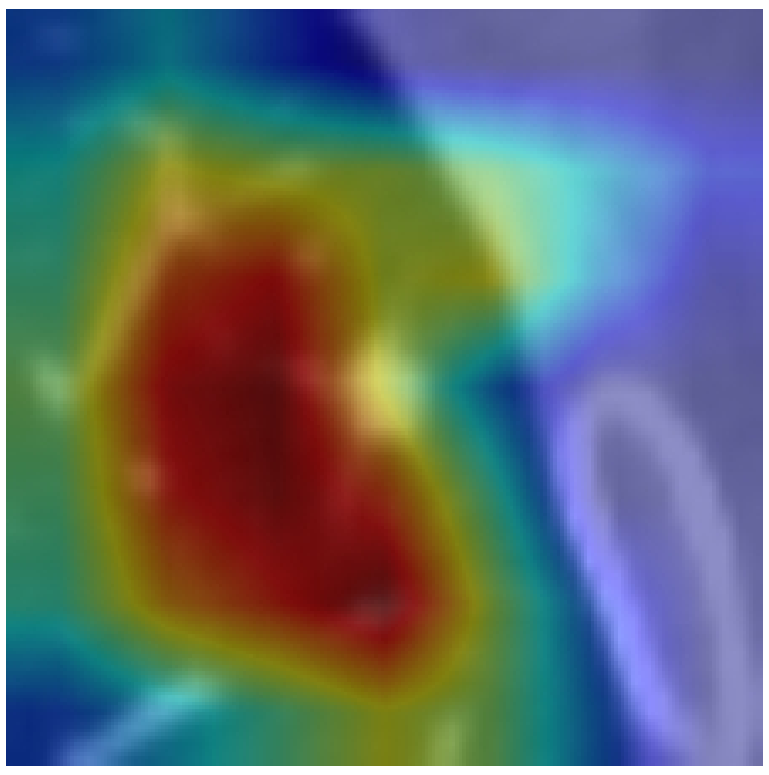

## Source information

Corresponding manuscript figure: Fig 8, Row 1
Sample ID: LIDC-IDRI-0129_825ffd6c_N06
Patient/Case ID: LIDC-IDRI-0129
Nodule ID/index: N06
Ground-truth label: Benign
View: Axial
Models: Single-view MedViT baseline and full proposed model
Grad-CAM target class: Model-predicted class
Checkpoint/model version: Best validation checkpoints used for final evaluation
Target layer: Predefined high-level feature layer of the axial-view encoder

# Fig 8. Case 2 unassembled Grad-CAM source images

**Original CT input** | **MedViT Grad-CAM** | **Proposed Model Grad-CAM**

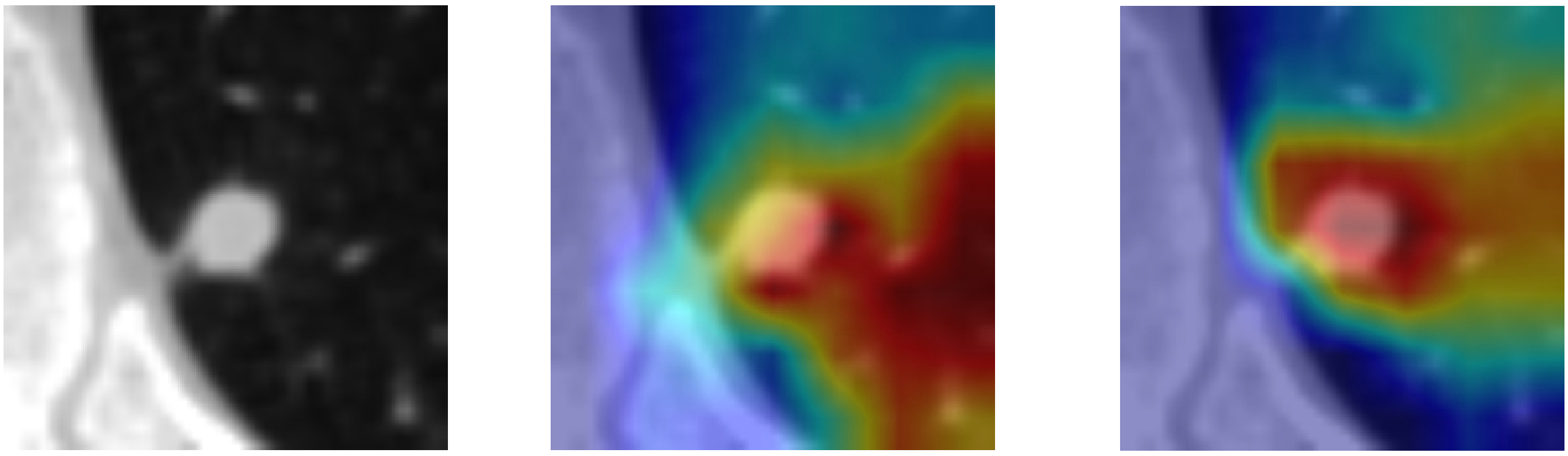

## Source information

Corresponding manuscript figure: Fig 8, Row 2
Sample ID: LIDC-IDRI-0264_033199ae_N02
Patient/Case ID: LIDC-IDRI-0264
Nodule ID/index: N02
Ground-truth label: Benign
View: Axial
Models: Single-view MedViT baseline and full proposed model
Grad-CAM target class: Model-predicted class
Checkpoint/model version: Best validation checkpoints used for final evaluation
Target layer: Predefined high-level feature layer of the axial-view encoder

# Fig 8. Case 3 unassembled Grad-CAM source images

**Original CT input**

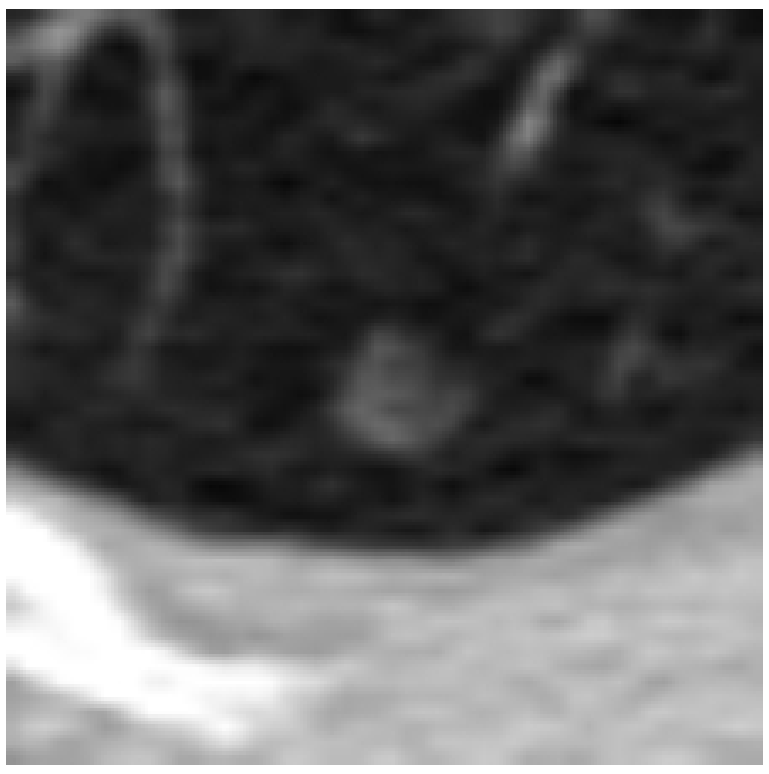

**MedViT Grad-CAM**

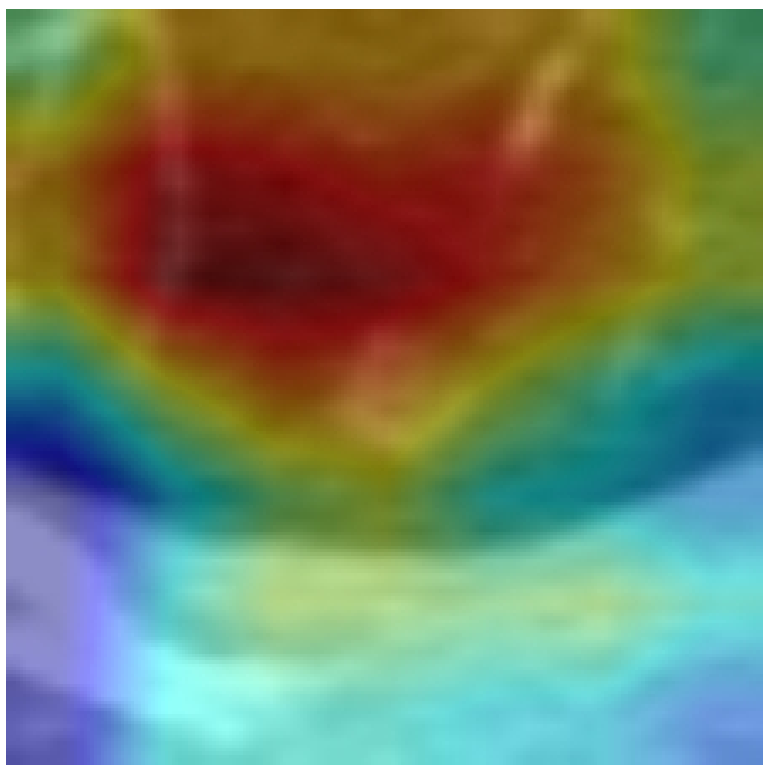

**Proposed Model Grad-CAM**

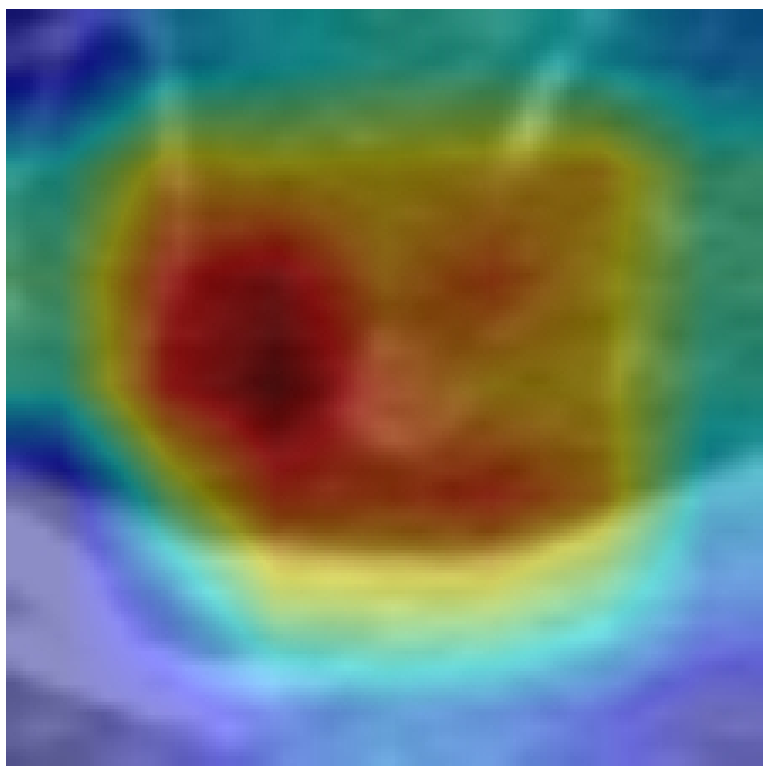

## Source information

Corresponding manuscript figure: Fig 8, Row 3
Sample ID: LIDC-IDRI-0413_ccd3a5a3_N03
Patient/Case ID: LIDC-IDRI-0413
Nodule ID/index: N03
Ground-truth label: Benign
View: Axial
Models: Single-view MedViT baseline and full proposed model
Grad-CAM target class: Model-predicted class
Checkpoint/model version: Best validation checkpoints used for final evaluation
Target layer: Predefined high-level feature layer of the axial-view encoder

# Fig 8. Case 4 unassembled Grad-CAM source images

**Original CT input** | **MedViT Grad-CAM** | **Proposed Model Grad-CAM**

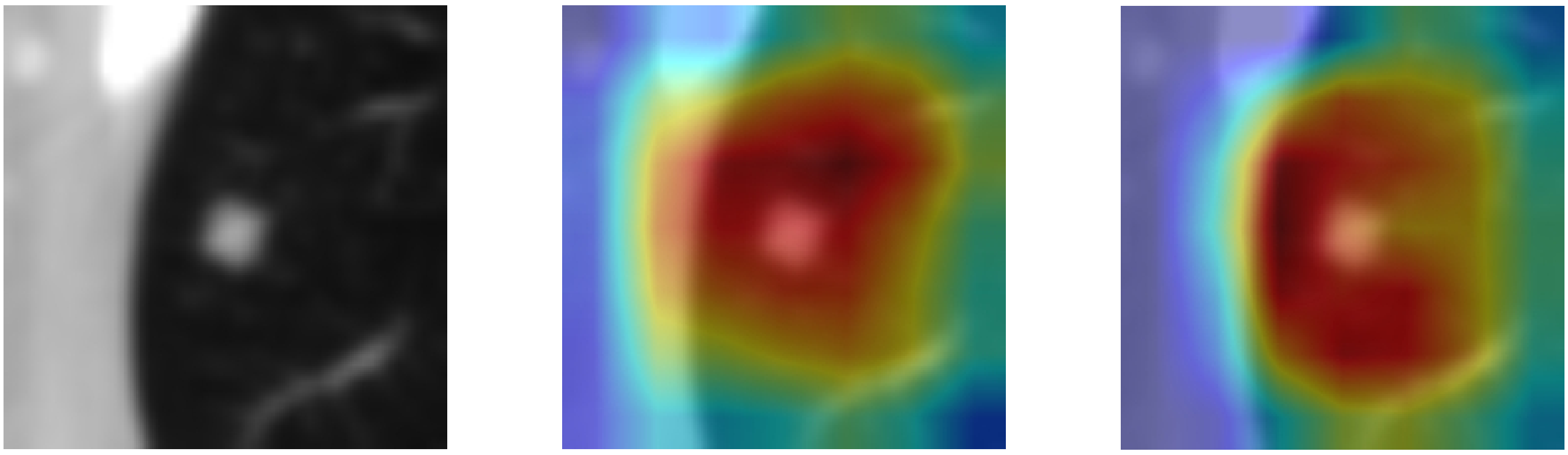

## Source information

Corresponding manuscript figure: Fig 8, Row 4
Sample ID: LIDC-IDRI-0583_f70514d7_N10
Patient/Case ID: LIDC-IDRI-0583
Nodule ID/index: N10
Ground-truth label: Benign
View: Axial
Models: Single-view MedViT baseline and full proposed model
Grad-CAM target class: Model-predicted class
Checkpoint/model version: Best validation checkpoints used for final evaluation
Target layer: Predefined high-level feature layer of the axial-view encoder

# Fig 8. Case 5 unassembled Grad-CAM source images

Original CT input | MedViT Grad-CAM | Proposed Model Grad-CAM

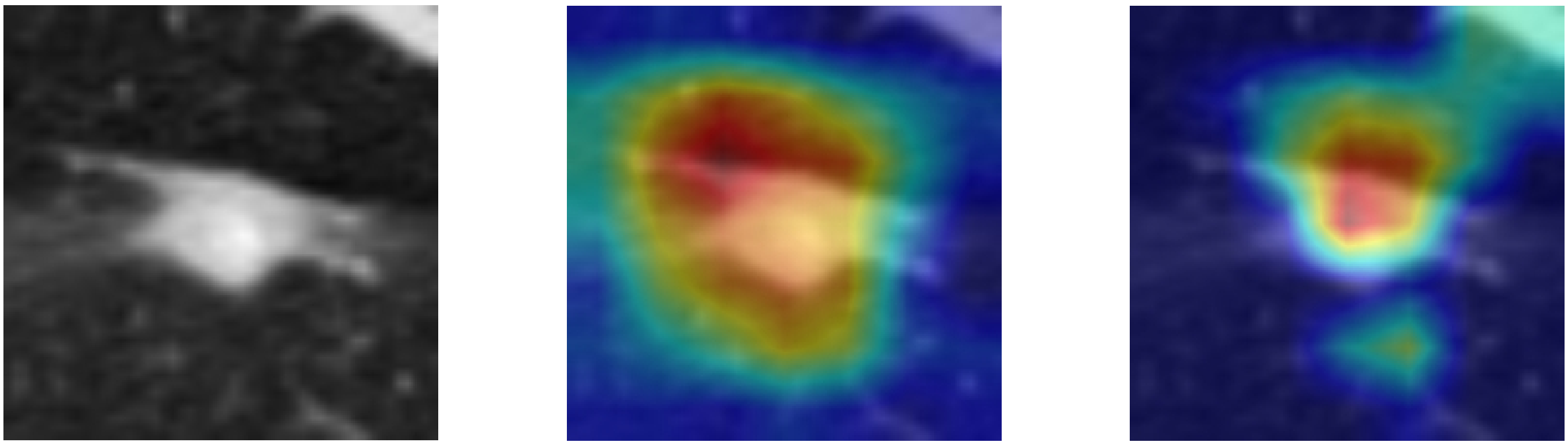

## Source information

Corresponding manuscript figure: Fig 8, Row 5
Sample ID: LIDC-IDRI-0720_24e56867_N02
Patient/Case ID: LIDC-IDRI-0720
Nodule ID/index: N02
Ground-truth label: Malignant
View: Axial
Models: Single-view MedViT baseline and full proposed model
Grad-CAM target class: Model-predicted class
Checkpoint/model version: Best validation checkpoints used for final evaluation
Target layer: Predefined high-level feature layer of the axial-view encoder
